# Combining directed acyclic graphs and the change-in-estimate procedure as a novel approach to adjustment-variable selection in epidemiology

**DOI:** 10.1186/1471-2288-12-156

**Published:** 2012-10-11

**Authors:** David Evans, Basile Chaix, Thierry Lobbedez, Christian Verger, Antoine Flahault

**Affiliations:** 1 Inserm UMR-S 707, Paris, France; 2 EHESP School of Public Health, Rennes-Sorbonne Paris Cité, Paris, France; 3 UPMC-Sorbonne Université, Paris, France; 4 Registre de Dialyse Péritonéale de Langue Française, Pontoise, France; 5 Nephrology Department, CHU Clemenceau, Caën, France

**Keywords:** Directed acyclic graph, Adjustment-variable selection, Change-in-estimate, Peritoneal dialysis

## Abstract

**Background:**

Directed acyclic graphs (DAGs) are an effective means of presenting expert-knowledge assumptions when selecting adjustment variables in epidemiology, whereas the change-in-estimate procedure is a common statistics-based approach. As DAGs imply specific empirical relationships which can be explored by the change-in-estimate procedure, it should be possible to combine the two approaches. This paper proposes such an approach which aims to produce well-adjusted estimates for a given research question, based on plausible DAGs consistent with the data at hand, combining prior knowledge and standard regression methods.

**Methods:**

Based on the relationships laid out in a DAG, researchers can predict how a collapsible estimator (e.g. risk ratio or risk difference) for an effect of interest should change when adjusted on different variable sets. Implied and observed patterns can then be compared to detect inconsistencies and so guide adjustment-variable selection.

**Results:**

The proposed approach involves i. drawing up a set of plausible background-knowledge DAGs; ii. starting with one of these DAGs as a working DAG, identifying a minimal variable set, S, sufficient to control for bias on the effect of interest; iii. estimating a collapsible estimator adjusted on S, then adjusted on S plus each variable not in S in turn (“add-one pattern”) and then adjusted on the variables in S minus each of these variables in turn (“minus-one pattern”); iv. checking the observed add-one and minus-one patterns against the pattern implied by the working DAG and the other prior DAGs; v. reviewing the DAGs, if needed; and vi. presenting the initial and all final DAGs with estimates.

**Conclusion:**

This approach to adjustment-variable selection combines background-knowledge and statistics-based approaches using methods already common in epidemiology and communicates assumptions and uncertainties in a standardized graphical format. It is probably best suited to areas where there is considerable background knowledge about plausible variable relationships. Researchers may use this approach as an additional tool for selecting adjustment variables when analyzing epidemiological data.

## Background

Adjustment-variable selection in epidemiology can be broadly grouped into background knowledge-based and statistics-based approaches. Directed acyclic graphs (DAGs) have come to be a core tool in the background-knowledge approach as they allow researchers to present assumed relationships between variables graphically and, based on these assumptions, to identify variables to adjust for confounding and other biases [1-3]. There is, however, no guarantee that the assumptions in such a prior DAG align with the patterns in the data. Stepwise selection based on p-values or the change-in-estimate are common statistics-based approaches [4]. In contrast to the background-knowledge approach, these allow patterns in the data to decide the final adjustment variables but risks in such data-driven approaches have been highlighted [5].

To our knowledge, only one methodological article in epidemiology to date has explicitly looked at combining background knowledge in DAGs with a statistical selection procedure for variable selection [6]. However, this article only considered stepwise deletion from an adjustment set defined from a prior DAG without checking whether the data supported the starting adjustment set. DAG-discovery algorithms, such as the PC and other algorithms in the TETRAD suite [7], combine background knowledge with statistical selection rules to discover DAG structures but they have proven controversial [8] and have not yet crossed over into epidemiological research. In fact, empirical articles [9-15] reporting DAGs for variable selection usually report only using prior DAGs, sometimes with subsequent stepwise deletion, but apparently without checking the starting assumptions against the data. Since the performance of these approaches depends on the appropriateness of the starting assumptions, a simple method for checking DAGs against the data may be valuable.

In this article, we propose an approach to adjustment-variable selection which aims to produce well-adjusted estimates for a given research question based on plausible DAGs which are also consistent with the data at hand, and to clearly communicate assumptions and uncertainties underlying the estimates in DAG format. It asks researchers to lay out prior assumptions about variable relationships in one or more prior DAGs, uses the change-in-estimate patterns in the data to refine and revise these DAGs, and presents the prior and final DAGs with corresponding estimates. The approach is based on recent theoretical results regarding confounding equivalence (c-equivalence) [16] and work on the collapsibility of estimates over different DAG structures [17]. To be pragmatic, the approach focuses on an exposure-outcome relationship of interest and uses regression models and the change-in-estimate procedure familiar to epidemiologists.

## Methods

### DAGs and minimally sufficient adjustment variable sets

In this article, we assume that the reader is familiar with the terminology of and rules for reading DAGs. There are now many introductions to DAGs for epidemiologists [ [1,2,17-20], annexe in [21]], including applications to specific areas of epidemiology [20,22]. DAGs are a graphical description of the joint probability distribution of a set of random variables, showing marginal and conditional (in)dependencies between variables [3,7,23,24]. We follow standard practice in epidemiology and give the arrows causal meaning, thereby interpreting a DAG as a causal diagram. We only address total associations in this article but the approach can be extended to direct and indirect effects based on graphical criteria for their identification [25-27].

DAGs allow the identification of the variable set or sets sufficient to adjust for confounding and other biases, based on the variable relationships shown. Greenland et al. [1] give conditions for this: a variable set is sufficient if i. there is no unblocked backdoor path joining the two variables which does not contain a variable in the set, and ii. there is no unblocked path joining the two variables induced by adjustment on the set which does not contain a variable in the set. This second condition means that if a collider is in the set and if adjusting on the collider unblocks the path between the two variables, then another variable on the path has also to be in the set to ensure that the path remains blocked. No variable in the set can be a descendant of the exposure or outcome [1]. (See [28] for a more recent formalization.) In practice, these conditions mean that the only unblocked paths joining exposure and outcome after conditioning on the adjustment variables can be mediating paths. A minimally sufficient adjustment set is a sufficient adjustment set which would no longer be sufficient if any variable were removed [2,29]. Minimally sufficient adjustment sets can be identified by manual [1,18] or computer [30,31] algorithms but a visual inspection is frequently sufficient.

### Drawing up prior DAGs

The first step is preparing a set of DAGs which encode prior, expert knowledge about variable relationships and show the major prior uncertainties. These DAGs should include

1. all measured variables considered relevant, including those routinely used for adjustment in the research area (e.g. sex) even if not thought *a priori* to be associated with other variables on the graph;

2. plausible proxy and measurement error relations;

3. plausible unmeasured parents with two or more children in the DAG; and

4. participation or selection variables conditioned upon during data-collection, including voluntary participation by subjects and restriction of the study to particular groups, such as hospitalized patients.

In most cases, more than one prior DAG will be needed to show the main uncertainties in variable relationships, including the presence or absence of arrows between variables, arrow direction, and the presence of unmeasured variables.

It is important to consider the source population of the data in preparing the prior DAG or DAGs. As much prior knowledge will come from research in other contexts, there will be cases when a researcher judges that an association between variables found in other studies do not apply in his or her dataset. For example, socioeconomic status may have an association with access to healthcare in systems with large out-of-pocket payments but not in well-functioning nationalized systems. In this case, the researcher needs to explain why he or she has chosen not to connect two variables which other researchers would connect, based on knowledge about source populations. Possible differences in source populations should also be borne in mind when revising the DAG, as discussed below.

### Using minimally sufficient adjustment sets to compare a DAG with data

For any given DAG, a researcher can identify the minimally sufficient adjustment set or sets for the effect of interest. Once done, he or she can identify the changes expected in this estimate when adjusting on different variable sets according to the DAG. To do this, we need to assume compatibility, faithfulness [32], and correct model specification. We also need to use a collapsible estimator (e.g. risk ratio (RR), risk difference (RD)), as the non-collapsible estimators (e.g. conditional odds ratio) can change upon adjusting on a variable which is strongly related with the outcome but is not, in fact, a confounder [33-35]. The RR and RD are therefore recommended and can now be readily estimated by regression [36-39].

Given the above, a collapsible effect estimate conditional on a minimally sufficient adjustment set will not change when estimated on this set plus the variables excluded from the set, provided that the excluded variables are not mediators (or ancestors or descendants of mediators) lying on an open path or colliders (or descendants of colliders) which, if conditioned upon, would open the path on which they lie. Conversely, a collapsible effect estimate conditional on a minimally sufficient adjustment set should change when estimated on this set minus any variable in the set. This allows a researcher to identify the change-in-estimate pattern implied by the DAG and so compare it with the observed pattern from the data.

Practically, we propose the following steps for this. Sample R-code is in Additional file 1 (web appendix):

1. Draw up the DAGs encoding prior, expert knowledge and the main prior uncertainties as described above and select an initial working DAG from this set (the most plausible DAG);

2. From the working DAG, identify a minimally sufficient adjustment set, S, for the effect of interest (A→Y);

3. Using a collapsible estimator, estimate A→Y conditional on S;

4. Re-estimate A→Y conditional on S plus each of the variables not included in S in turn (“add-one pattern”);

5. Plot each estimate on a single graph, thereby showing differences in the estimates between the models;

6. Repeat steps 4 and 5 but deleting each variable in turn from S (“minus-one pattern”);

7. Determine whether the add-one and minus-one patterns found are consistent with the working DAG;

8. If the patterns are consistent with the working DAG, check to see if any of the other prior DAGs give the same expected patterns. Take all prior DAGs with consistent patterns as the revised working DAGs and move to step 11;

9. If the patterns are not consistent with the working DAG, check to see if any of the other prior DAGs imply the patterns as observed. Take all such consistent prior DAGs as the revised working DAGs and move to step 11;

10. If the patterns are not consistent with the working DAG or with any of the other prior DAGs, undertake an *ad hoc* revision (see web appendix) to create a new working DAG;

11. Repeat steps 2 to 11 for each revised working DAG, moving to step 12 when there are no inconsistent add-one and minus-one patterns;

12. Present the prior and all final DAGs with corresponding effect estimates.

**Figure 1:**
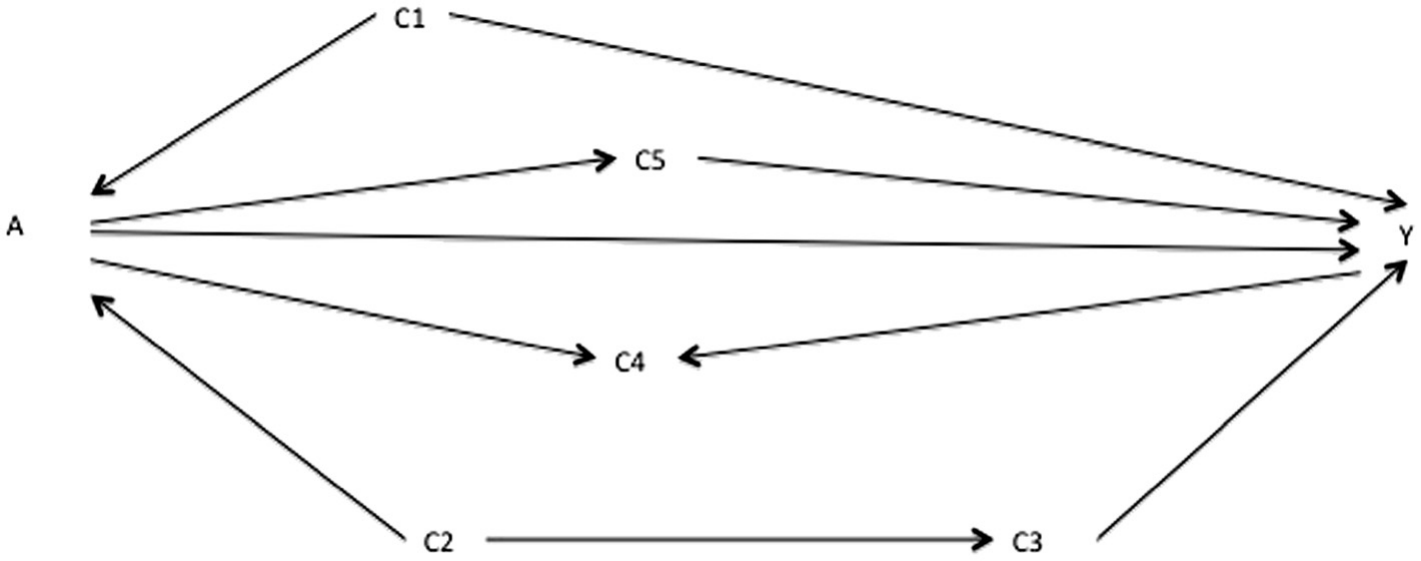
Directed acyclic graph showing putative relationships between variables A, Y, C1, C2, C3, C4, and C5.

The key to step 7 is recognizing when the observed patterns are consistent with the patterns implied by the DAG. If S is minimally sufficient, the add-one pattern is consistent if the only meaningful changes arise when conditioning on mediators lying on open paths from A to Y or when conditioning on colliders which open a path from A to Y. All variables in S should show meaningful minus-one changes, but this may not always be the case in practice because of incidental cancellations (see Discussion). Once familiar with the rules of DAGs, it is straightforward for a researcher to identify the expected changes for any adjustment set for a given DAG: for example, if adjusting on {C_1_,C_3_} in Figure 1, the implied add-one pattern is no change for C_2_ and a change for C_4_ and C_5_. The implied minus-one pattern is a change for C_1_ and C_3_.

Importantly, DAGs will commonly have more than one minimally sufficient adjustment set. In this case, the researcher should also compare the effects estimated on each minimally sufficient set in steps 8 and 9 above. These adjusted effect estimates should not differ, meaning that any observed differences can help distinguish between the different working DAGs in these steps.

### Defining a meaningful change

A key decision is defining the change in the estimate sufficient to warrant reviewing the DAG. The first issue here is the size of the change. For this, a researcher could choose to follow (and defend) the commonly used threshold of a 10% relative difference in the starting estimate [4,40]. Although standard practice in epidemiology, the relative nature of this rule means that the chance of declaring a change meaningful will differ with the magnitude of the starting estimate (see empirical example below). An alternative to consider is therefore using absolute change, which, given arguments that the absolute RD is particularly relevant to decision-making [37], also has the benefit of allowing a researcher to determine the threshold based on judgements of clinical or public-health relevance [36]. For example, the threshold could be the difference in mortality or in non-persistence to a prescribed treatment which would warrant a clinical or public-health reaction. If no consensus threshold is available for certain questions, the researcher will need to propose (and defend) a reasonable value. Although arbitrary, this approach has the benefit of transparently communicating the decision rule and its rationale to other researchers, who can adopt or challenge it. The choice of estimator and of the meaningful threshold therefore clearly depend on the research question but should be defined and justified before analysis.

The second issue here is variability in the change in estimate because of sampling error or other problems such as unstable models. In this case, a researcher may inappropriately revise (or not revise) a prior DAG because the observed patterns have failed to align with the patterns in the source population by chance. We note, however, that this is the case for the change-in-estimate procedure as currently practised as it only uses the point estimate change to guide covariable selection.

To incorporate variability into the proposed approach, we suggest estimating the expected proportion of times the add-one and minus-one patterns would lead to a revision of the DAG under resampling and using this information in a sensitivity analysis. This can be done by bootstrap, calculating the proportion of resampled estimates lying beyond the meaningful change threshold for each variable during the add-one and minus-one steps. The researcher should report these proportions for the prior working and final DAGs. We also suggest undertaking a sensitivity analysis by revising the prior working DAG considering only variables with >50% of resampled add-one changes outside the meaningful threshold as showing meaningful changes. Although this will mean presenting several final DAGs, it has the merit of communicating uncertainty in the assumptions used for the final models. In contrast, for the minus-one step we suggest only reporting the proportion of resampled estimates without undertaking the sensitivity analysis for the reasons outlined in the Discussion.

There are two important caveats here. First, the proposed 50% cut-off for the add-one changes is arbitrary and further studies should explore the performance of different cut-off values. Second, inflated variance estimates because of unstable regression models (e.g. small sample size, collinearity) would also lead to a high estimated variability of the changes, highlighting the importance of routine model checking in the approach.

### Reviewing the DAG

An important issue in reviewing the working DAG (steps 7 to 10 above) is that, as numerous DAGs can be constructed around the same variables, there is a risk of revision *a posteriori* to fit the observed empirical pattern. To mitigate this, we suggest first addressing the prior uncertainties as represented by the set of alternative, prior DAGs. If these DAGs do not include a graph consistent with the observed patterns, the researcher will need to consider other possible misspecification of confounding, mediating, and collision pathways, measurement error, and bias amplification as outlined in the Results. A structured approach to working through these possibilities is in Additional file 1 (web appendix). However, given the risk of *post hoc* fitting the DAG to the data at this stage, the researcher should state that none of the prior DAGs was consistent with the observed patterns. Note that model misspecification, another reason to consider, is not addressed in this article for reasons of space. As noted, usual methods for model checking clearly apply.

## Results

We now run through a theoretical example to illustrate the approach before presenting an empirical example from clinical epidemiology.

### Confounding, mediation, collision

**Figure 2:**
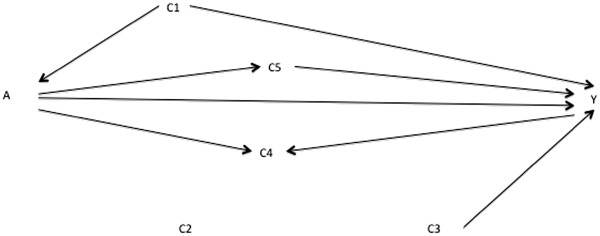
Directed acyclic graph showing alternative putative relationships between variables A, Y, C1, C2, C3, C4, and C5.

**Figure 3:**
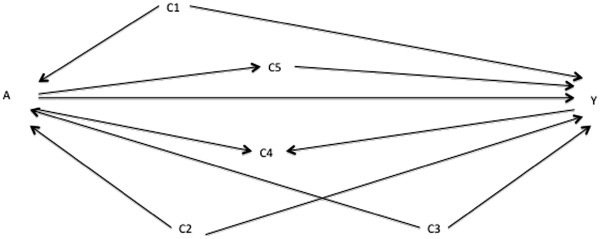
Directed acyclic graph showing one set of alternative putative relationships between variables A, Y, C1, C2, C3, C4, and C5.

**Figure 4:**
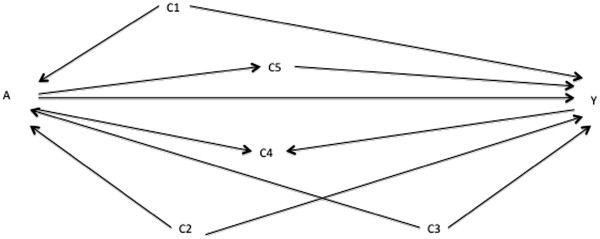
Directed acyclic graph showing another set of alternative putative relationships between variables A, Y, C1, C2, C3, C4, and C5.

**Figure 5:**
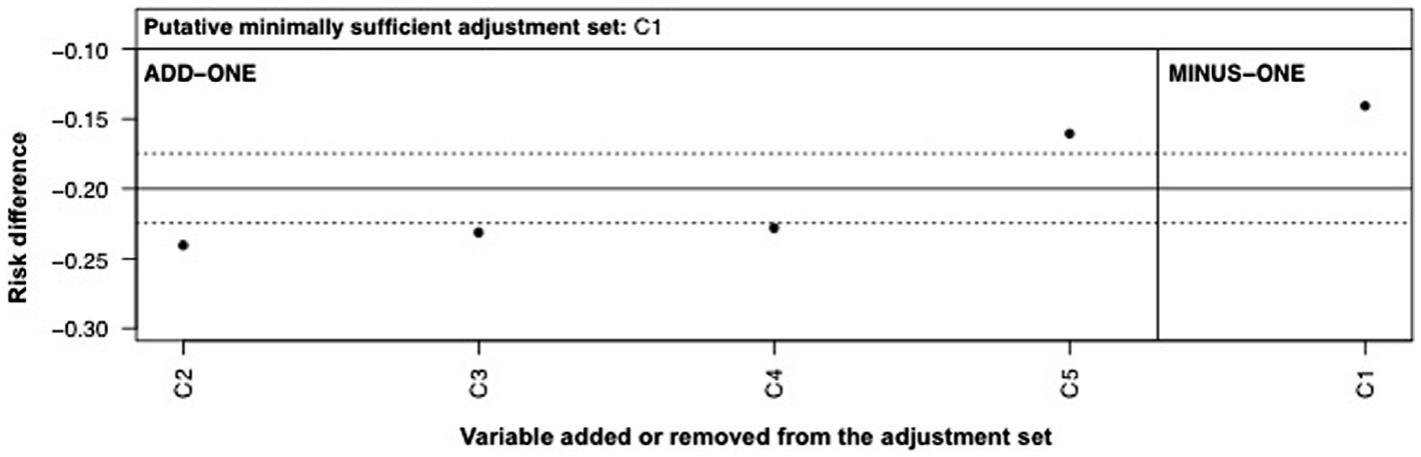
**Add**-**one and minus**-**one patterns for a starting adjustment**-**variable set of{C**_**1**_**}based on DAG in Figure**2, **taking the associations in the DAG in Figure**1**as the unknown best working DAG.** The solid horizontal line is the RD estimate adjusted on the putative minimally sufficient set {C_1_}. The dashed horizontal lines are the pre-defined meaningful change thresholds in the RD estimate. The add-one section shows the RD upon adding each variable listed to the adjustment-variable set in turn. The minus-one section shows the RD upon removing each variable listed from the adjustment-variable set in turn.

Take the (as yet unknown) best-working DAG in Figure 1, the prior DAG in Figure 2 as the preferred initial working DAG, and the DAGs in Figures 1, 3, and 4 as prior alternative DAGs. These figures are also available in Additional file 2 in slide format to follow the changes by flicking back and forth between figures. From Figure 2, a researcher identifies a putative minimally sufficient adjustment set of {C_1_}. The implied add-one pattern for Figure 2 when adjusting on {C_1_} is a change for C_4_ and C_5_ and no change for C_2_ or C_3_; the implied minus-one pattern is a change for C_1_. He or she estimates the A→Y effect adjusted on {C_1_} and the add-one and minus-one patterns. Graphing this (step 5 above) gives a pattern as in Figure 5, where the dotted horizontal lines represent the pre-defined threshold for a meaningful change. The changes on adding C_4_ and C_5_ and for removing C_1_ are consistent with Figure 2. In contrast, the changes for adding C_2_ and C_3_ are not consistent with Figure 2, flagging the need to reconsider them.

During preparation of the prior DAGs, our researcher flagged the possible confounding pathways in Figures 1 or 3 and C_2_ as a collider in Figure 4. Both Figures 1 and 4 have the same implied add-one and minus-one patterns when adjusting on C_1_ only, namely add-one changes for C_2_, C_3_, C_4_, and C_5_ and minus-one changes for C_1_. These are consistent with Figure 3. The implied patterns for Figure 4 when adjusting on C_1_ only are add-one changes for C_2_, C_4_, and C_5_; no add-one change for C_3_; and a minus-one change for C_1_. These do not correspond to those observed in Figure 5 (the add-one pattern should not change for C_3_). Consequently, the researcher can discount the DAG in Figure 4 and focus on Figures 1 and 3.

The researcher should reapply the above steps to each of Figures 1 and 3. In Figure 3, the minimally sufficient adjustment set is {C_1_,C_2_,C_3_}. The implied patterns adjusting on this set is an add-one change for C4 and C5 and a minus-one change for C_1_, C_2_, and C_3_. As Figure 1 is the still unknown best working DAG, the observed pattern will have no minus-one change for C_2_ and C_3_. In contrast, re-running the steps on Figure 1 will obviously give consistent add-one and minus-one patterns. This favours Figure 1. The researcher can go further, noting that both {C_1_,C_2_} and {C_1_,C_3_} are minimally sufficient adjustment sets in Figure 1. The effect estimate adjusted on each of these sets does not change, consistent with Figure 1 as the final working DAG based on these prior starting DAGs.

Alternatively, the researcher may have pre-identified uncertain mediation paths involving C_2_ and C_3_, for example a single mediating path (A→C_2_→C_3_→Y) or two separate mediating paths (A→C_2_→Y and A→C_3_→Y) (not shown but easily constructed by replacing A←C_2_ with A→C_2_ in Figures 1 and 3 and A←C_3_ by A→C_3_ in Figure 3). The same approach as for the confounding scenarios will help distinguish between these, although, as discussed below, background knowledge is required to decide on the confounding vs. mediating direction of the arrows.

### Measurement error

**Figure 6:**
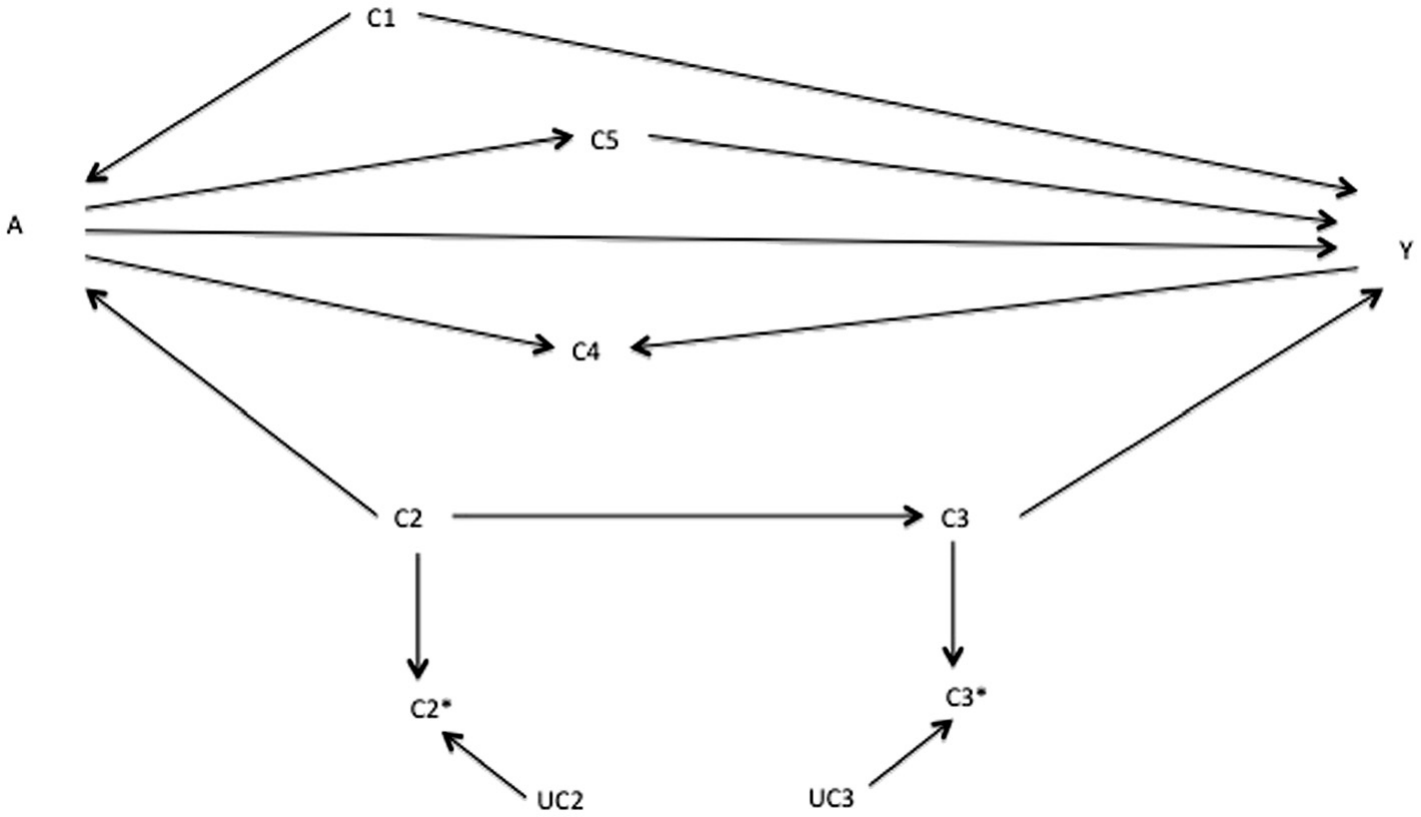
Directed acyclic graph showing alternative putative relationships between variables A, Y, C1, C2, C3, C4, and C5 in which C2 and C3 are measured with error (measured variables are C2* and C3* and variables affecting their measurement are UC2 and UC3).

Measurement error can also cause an estimate to change when adding or deleting variables to or from the adjustment set, even though this would not be the case had the variables been measured perfectly. To see why, consider Figure 6, which is Figure 1 with measurement error of C_2_ and C_3_. Following [41], we define C* as the measured variable, and U_C_ as representing all factors affecting measurement of C. Adjusting on C_2_* only partially blocks A←C_2_→C_3_→Y at C_2_; similarly, adjusting on C_3_* only partially blocks this pathway at C_3_; consequently the estimate adjusted on {C_1_,C_2_*} will not equal that adjusted on {C_1_,C_2_*,C_3_*} even though they would have been the same if we could have adjusted on {C_1_,C_2_} and {C_1_,C_2_,C_3_}.

**Figure 7:**
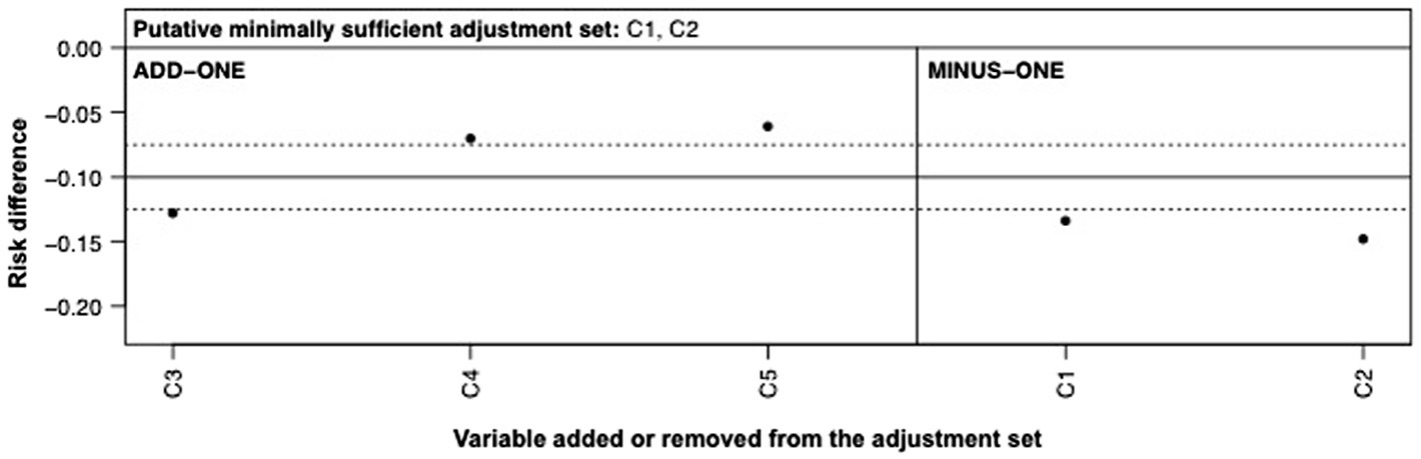
**Add**-**one and minus**-**one patterns for a starting adjustment**-**variable set of** {**C**_**1**_, **C**_**2**_} **based on DAG in Figure**1, **taking the associations in the DAG in Figure**6**as the unknown best working DAG.** Note that the variables listed as C_2_ and C_3_ are actually these variables measured with error, i.e. C_2_* and C_3_* in Figure 6. The solid horizontal line is the RD estimate adjusted on the putative minimally sufficient set {C_1_}. The dashed horizontal lines are the pre-defined meaningful change thresholds in the RD estimate. The add-one section shows the RD upon adding each variable listed to the adjustment-variable set in turn. The minus-one section shows the RD upon removing each variable listed from the adjustment-variable set in turn.

To see how measurement error fits into the proposed approach, consider the case of Figure 6 as the (unknown) best working DAG, Figure 1 as a researcher’s initial working prior DAG, and measurement error of C_2_ and C_3_ in Figure 6 as an alternative prior DAG. Running through the above steps on Figure 1 using a minimally sufficient adjustment set of {C_1_,C_2_} will give add-one and minus-one patterns as in Figure 7. These are inconsistent for C_3_ in Figure 1, since adding C_3_ to the {C_1_,C_2_} adjustment set should not change the estimate. In contrast, this pattern is consistent with the measurement error in Figure 6. Although, intuitively, the “best” adjustment set is expected to be {C_1_,C_2_*,C_3_*}, adjusting on a mismeasured confounder may increase bias under certain conditions [42,43] such as the presence of a qualitative interaction between exposure and confounder if the confounder is binary [43]. Even in conditions for which adjustment on {C_1_,C_2_*,C_3_*} will be bias reducing, arguably common in epidemiological research [43-45], this will not be a sufficient adjustment set as it only partially blocks the A←C_2_→C_3_→Y pathway. Regardless of the direction of the bias, the proposed change-in-estimate approach should flag the need to review the associations involving the mismeasured variables in the DAG.

### Bias amplification

Recent work has shown that residual bias can be amplified by adjustment on instrument-like variables [46,47], a finding which, although its quantitative relevance is still under debate [48,49], has potentially major implications for adjustment-variable selection in epidemiology. Such bias amplification can also lead to a change in the effect estimate when adjusting on different variable sets, so researchers should consider it when reviewing a DAG based on the add-one and minus-one patterns. Note that “instrument-like” refers to variables which are strong predictors of the exposure but can be also associated with the outcome (see [46] for detailed discussion and estimate of the ratio of two associations). Confounders can therefore be instrument-like, depending on the relative strength of their relationships with the exposure and the outcome. This is not to be confused with standard instrumental variables which, by definition, are associated only with the exposure and which have bias-reducing properties in appropriate analyses (see [50] for this) and bias-amplifying effects in other analyses [46].

**Figure 8:**
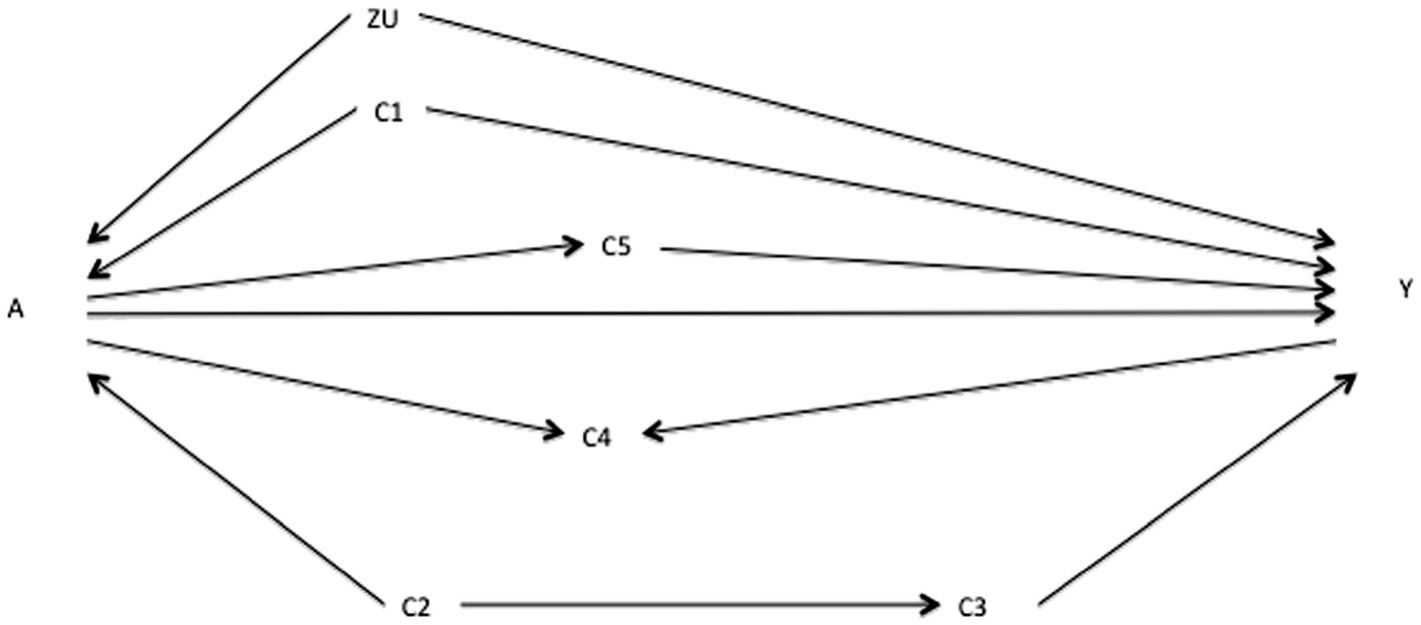
Directed acyclic graph showing alternative putative relationships between variables A, Y, C1, C2, C3, C4, C5, and an unmeasured variable ZU.

Consider Figure 1 as a prior DAG, Figure 8 as the unknown best working DAG, and major residual confounding, shown by the pathway A←Z_U_→Y in Figure 8, as a prior uncertainty. In the absence of residual confounding (Figure 1), a collapsible estimate adjusted on {C_1_,C_2_}, {C_1_,C_3_}, and {C_1_,C_2_,C_3_} should not differ. However, with residual confounding (Figure 8), these estimates will differ because C_2_ and C_3_ have different “instrument strengths” (i.e. relative to C_3_, C_2_ is more strongly associated with the exposure A) and so amplify the residual bias differently [16]. Consequently, a researcher starting with a minimally sufficient adjustment set of {C_1_,C_2_} (based on Figure 1) will find add-one and minus-one patterns similar to those shown in Figure 7. These patterns are inconsistent with Figure 1 but are consistent with the alternative DAG in Figure 8. The question again becomes which adjustment set to choose to minimize bias. Until further theoretical and simulation work is available on bias amplification, a conservative strategy is to adjust on {C_1_,C_3_}, as C_3_ should be a weaker instrument than C_2_, but also to present the estimate adjusted on {C_1_,C_2_} and {C_1_,C_2_,C_3_}.

### Presenting more than one final DAG

In many instances, the researcher will need to present more than one final DAG with implied add-one and minus-one patterns consistent with the patterns observed. Sometimes the adjusted estimate will be the same as the DAGs imply the same minimally sufficient adjustment set. An example is removing the C_5_→Y arrow and adding a C_5_←C_3_ arrow in Figure 2. This DAG has similar implied patterns as the current Figure 2 and so, if matching the observed patterns, both would need to be presented amongst the final DAGs. The minimally sufficient adjustment set in both is {C_1_} and so the adjusted effect estimate will be the same. However, in some cases the minimally sufficient adjustment sets will be different, so that an estimate for each DAG will need to be presented. One example of this involves the confounding vs. mediating pathways mentioned above, if both types of relationship were identified as plausible during the preparation of the prior DAGs (e.g. the DAG in Figure 4 and the DAG created by replacing A←C_2_→Y with A→C_2_→Y in Figure 4).

### Empirical example

We now consider an empirical example to illustrate the approach. We compare mortality 5 years after peritoneal-dialysis (PD) initiation amongst patients with polycystic kidney disease (PKD) versus other nephropathies, using data from the French Language Peritoneal Dialysis Registry (RDPLF) (details in Additional file 1 (web appendix); see also [51] for background). We estimate the RD by linear regression with robust standard errors [52] and use a ±0.01 absolute change in the point estimate of the RD as meaningful, considering that difference of this magnitude in the cumulative incidence of death would warrant attention from clinical or public health decision-makers. To compare the absolute with relative scales, we also show a ±10% change in the RD. We calculated the proportion of estimates lying outside the ±0.01 absolute change threshold on resampling using 2000 non-parametric bootstrap samples.

**Figure 9:**
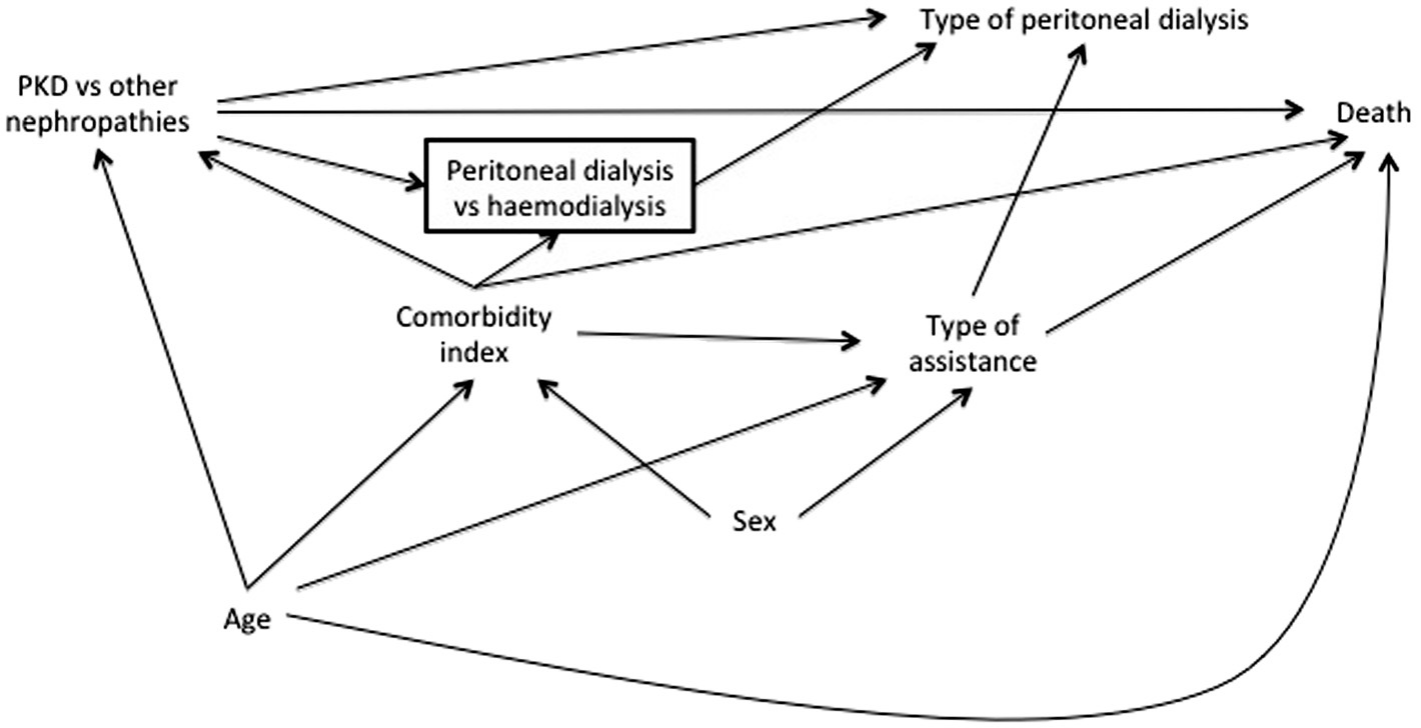
Directed acyclic graph showing prior assumptions about relationships between variables in the empirical example.

**Figure 10:**
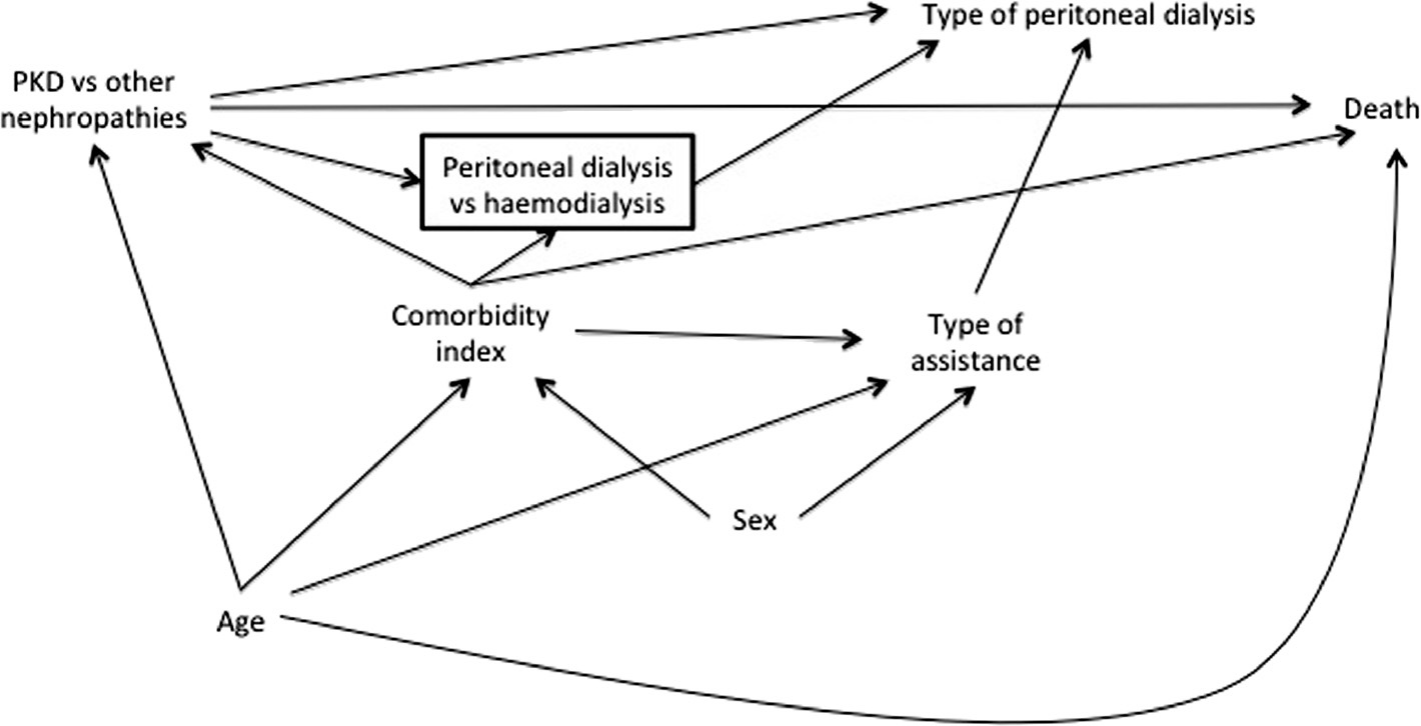
Directed acyclic graph showing prior uncertainty about variable relationships in the empirical example (absence of Type of Assistance -> Death arrow).

**Figure 11:**
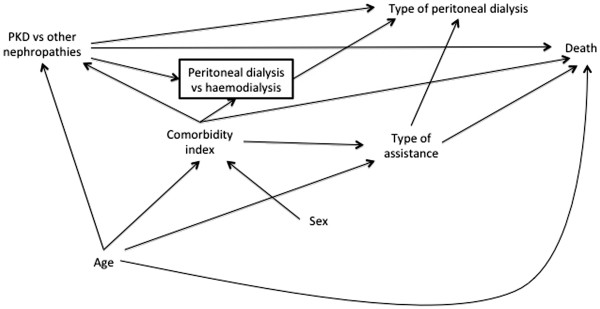
Directed acyclic graph showing prior uncertainty about variable relationships in the empirical example (absence of Sex -> Type of Assistance).

**Figure 12:**
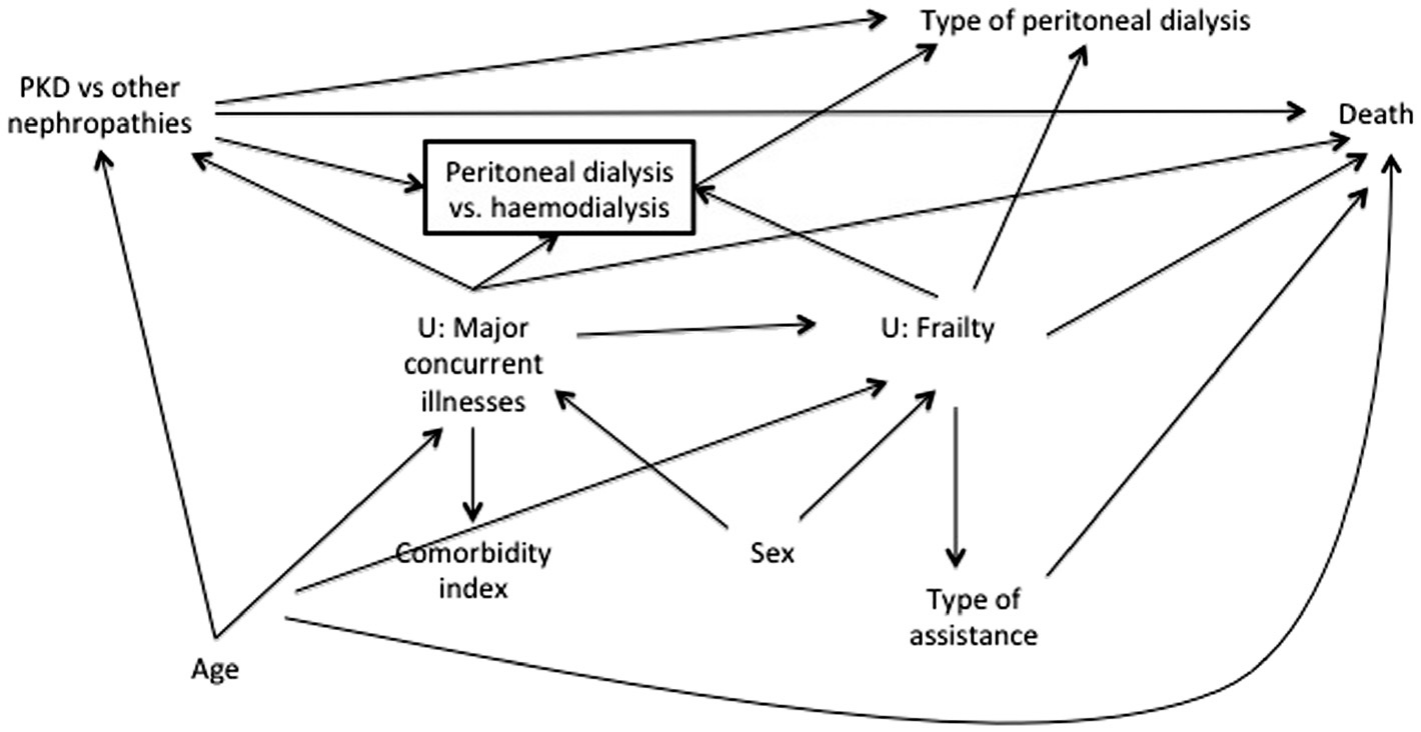
Directed acyclic graph showing prior uncertainty about variable relationships in the empirical example (showing Comorbidity index and Type of assistance as proxy variables for Major concurrent illnesses and Frailty, respectively).

The DAG in Figure 9 illustrates prior assumptions regarding variable relationships. Type of peritoneal dialysis refers to the two modalities of treatment, namely continuous ambulatory peritoneal dialysis and automated peritoneal dialysis. The other variables are self-explanatory. Figure 9 shows, for example, that we assume that *Type of peritoneal dialysis* and *Sex* have no direct association with *Death* and that both *PKD vs. other nephropathies* and *Comorbidity index* are associated with the *Peritoneal dialysis vs. haemodialysis* participation variable. The square around this latter variable shows that it has been conditioned upon during data collection, since only PD patients are included in the registry. Our prior uncertainties are absence of the *Type of assistance*→*Death arrow* (Figure 10), absence of the *Sex*→*Type of assistance* arrow (Figure 11), and whether *Comorbidity index* and *Type of assistance* are better considered as proxies for two unmeasured variables, *Major concurrent illnesses* and *Frailty*, respectively (Figure 12). In this last case, we consider *Frailty* also to be associated with the *Peritoneal dialysis vs. haemodialysis* collider and with *Death*.

**Figure 13:**
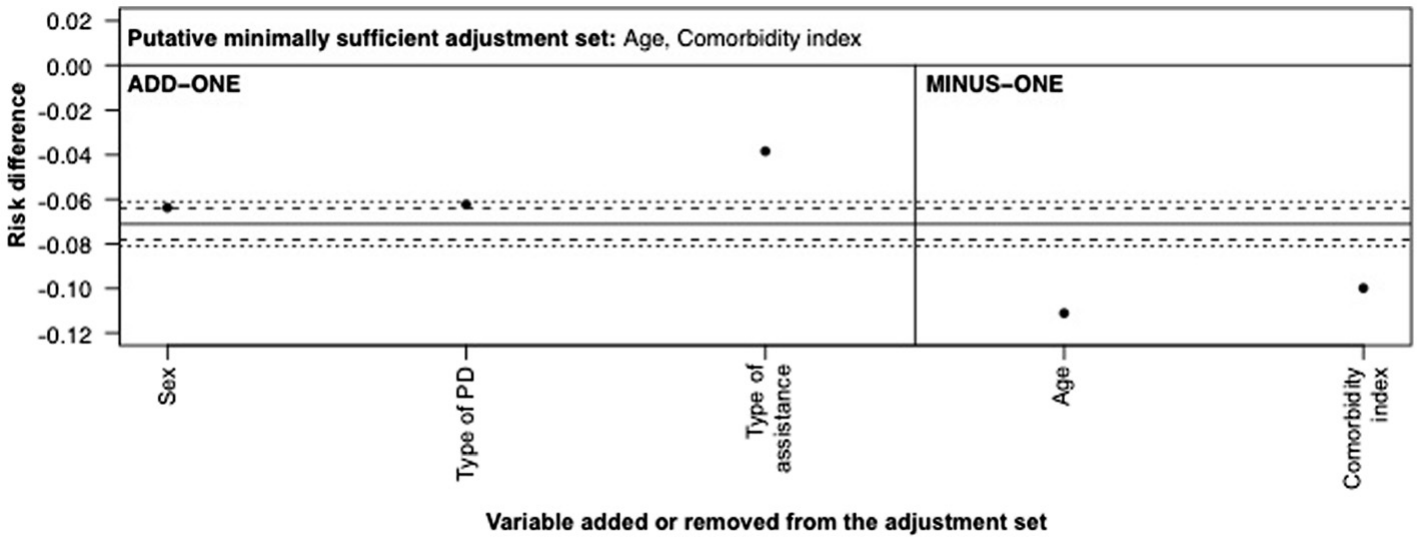
**Add**-**one and minus**-**one patterns for a adjustment**-**variable set of{*Age*, *Comorbidity index*}based on DAG in Figure**9 The solid horizontal line is the RD estimate adjusted on this set. The dotted horizontal lines are the pre-defined meaningful change thresholds for an absolute change of ± 0.01 in the RD. The dashed horizontal lines are a relative change of ±10% of the starting RD. The add-one section shows the RD upon adding each variable listed to the adjustment-variable set in turn. The minus-one section shows the RD upon removing each variable listed from the adjustment-variable set in turn.

There is only one minimally sufficient adjustment set in the prior DAG (Figure 9), simply {*Age*, *Comorbidity index*}. Figure 13 shows the add-one and minus-one patterns for this adjustment set. The dotted lines are the ±0.01 threshold; the dashed lines are the 10% relative change in the RD. The add-one pattern shows a meaningful change for *Type of assistance* (i.e. lies outside of the dotted line in Figure 13), inconsistent with the implied pattern from Figure 9, whereas the minus-one pattern shows a meaningful change for both variables in the set, consistent with Figure 9. The proportions of bootstrapped estimates lying outside of the meaningful threshold are in Table 1: only *Type of assistance* had >50% of the add-one estimates outside of the meaningful threshold.

**Table 1:** **Percentage of bootstrapped risk difference estimates representing a meaningful change** (± **0**.**01 change**) **for each variable in the empirical example**

			
For Figure 13			
Add-one variables		Minus-one variables	
Sex	28.4%	Age	95.3%
Type of peritoneal dialysis	37.1%	Comorbidity index	98.6%
Type of assistance	99.6%		
For Figure 14			
Add-one variables		Minus-one variables	
Type of peritoneal dialysis	15.2%	Age	38.3%
		Comorbidity index	58.8%
		Sex	75.9%
		Type of assistance	100.0%

We therefore need to review the DAG, focusing on *Type of assistance*. Looking at the prior uncertainties, dropping the *Type of assistance*→*Death* (Figure 10) or the *Sex*→*Type of assistance* arrows (Figure 11) does not change the implied patterns compared with Figure 9. In contrast, specifying the proxy relations in Figure 12 changes the adjustment set. (Note that there is no sufficient adjustment set (of measured variables) according to this DAG as the paths *PKD vs. other nephropathies*←*Major concurrent illnesses*→*Death*, *PKD vs. other nephropathies*←*Major concurrent illnesses*→*Frailty*→*Death*, PKD vs. other nephropathies←Major concurrent illnesses→Peritoneal dialysis vs. haemodialysis←Frailty→Death, and *PKD vs. other nephropathies*→*Peritoneal dialysis vs*. *haemodialysis*←*Frailty*→*Death* remain partially open at *Major concurrent illnesses* and *Frailty*.) The implied add-one pattern for a starting adjustment set of {*Age*, *Comorbidity index*} in Figure 12 is therefore a meaningful change for *Type of assistance*, *Sex*, and *Type of peritoneal dialysis*.

**Figure 14:**
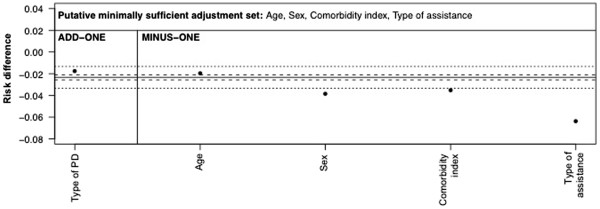
**Add**-**one and minus**-**one patterns for a adjustment**-**variable set of *{Age***, ***Comorbidity index***, ***Type of assistance***, ***Sex} *based on DAG in Figure**12 The solid horizontal line is the RD estimate adjusted on this set. The dotted horizontal lines are the pre-defined meaningful change thresholds for an absolute change of ± 0.01 in the RD. The dashed horizontal lines are a relative change of ±10% of the starting RD. The add-one section shows the RD upon adding each variable listed to the adjustment-variable set in turn. The minus-one section shows the RD upon removing each variable listed from the adjustment-variable set in turn.

Now using Figure 12 as our revised working DAG, the best adjustment set is {*Age*, *Comorbidity index*, *Type of assistance*, *Sex*}. The last three variables are included as descending or ascending proxies of the two unmeasured variables. We did not include *Type of peritoneal dialysis* in this set as its net bias-reducing effect is not clear, noting that it will contributed to partially conditioning on the unmeasured *Frailty* variable but will also open biasing pathways, e.g. *PKD vs. other nephropathies*→*Type of peritoneal dialysis*←*Frailty*→*Death*. The RD adjusted on the final set did not show a meaningful change in the add-one pattern (proportion of bootstrapped estimates outside of threshold <50% shown in Table 1) and the minus-one pattern showed a meaningful change for all adjustment variables except *Age* (Figure 14). *Age* also had <50% of bootstrapped estimates lying outside of the meaningful threshold (Table 1). We maintain *Age* in the adjustment set as this pattern is coherent with the DAG, since the other adjustment variables, *Comorbidity index* and *Type of assistance*, may already condition effectively on *Age* owing to a strong correlation. However, we note that *Age* may be dropped if it improves the efficiency of the estimate (see [6]). We would therefore present our prior working DAG (Figure 9) with an RD of −0.07 (95%CI: -0.14, 0.00) and our final working DAG (Figure 12) with an RD of −0.02 (95%CI: -0.10, 0.05).

As an aside, Figures 13 and 14 show the difference between using relative and absolute scales as the threshold for a meaningful change. In Figure 13, the starting RD is −0.07 and so the width of the relative change (dashed lines) is close to that of the absolute change (dotted lines). In Figure 14, the starting RD is considerably smaller, at −0.02, and so the width of the relative change is much smaller than that of the absolute change.

## Discussion

We have presented an approach to selecting adjustment variables which combines prior knowledge expressed in a DAG with results from analysis of the data. The approach is pragmatic in that it focuses only on the effect of interest (also emphasized by others [5]); uses regression models and the change-in-estimate procedure familiar to epidemiologists; and can incorporate real-data problems such as measurement error and residual bias. It aims at producing a plausible, best working DAG or set of DAGs for a given research question, given the data at hand, and at communicating the assumptions underlying variable selection in the initial and final models using a standardized, graphical form [3]. The approach also communicates the uncertainties in the assumptions in the final models by presenting all the DAGs identified by the researcher which are consistent with the observed change-in-estimate patterns. This aims to help other research teams to focus on the areas of uncertainty and corroborate or refute the DAGs, based on the analysis of different datasets in an iterative way.

The approach depends on recent theoretical work on c- (confounding-) equivalence [16] and collapsibility of estimates over different DAG structures [17]. Pearl and Paz [16] have developed conditions for c-equivalence which apply to any subsets of the variables in a DAG. Our approach uses two of their results: that all sufficient adjustment sets are c-equivalent and that failure to find c-equivalence of putative sufficient adjustment sets rules out a DAG implying such c-equivalence [3]. The approach also uses Pearl and Paz’s insights into bias amplification, in which they note that bias amplification will lead to changes in associations conditional on different variables even if the variables block the same path. In a recent, detailed review of collapsibility (i.e. equivalence) of different estimators over different DAGs [17], Greenland and Pearl noted that regression coefficients may be used to check collapsibility over different covariable sets, an approach which we develop here for applied work.

To our knowledge, only one other article in the epidemiology literature to date has looked at adjustment variable selection by explicitly combining DAGs and a statistical selection procedure [6]. This article addressed deletion of variables from an adjustment set defined from a prior DAG using the change-in-estimate procedure, but considered only odds ratios from simulations of case–control studies and explicitly excluded colliders. Our approach is therefore broader as it addresses whether the data support the initial DAG which defines the starting adjustment set, applies to any collapsible estimator, and covers the range of possible relationships between variables. Interestingly, this article found largest bias (using simulated data) when including covariables associated only with the outcome in the adjustment set and suggested that non-collapsibility of the odds ratio may have been involved [6]. This reinforces our insistence on collapsible estimators.

The proposed approach has some potential advantages over other variable-selection methods. It can reduce the “black-box” nature of using the p-value or the change-in-estimate alone to select variables, as it lays out the rationale for adjustment-variable choice graphically. It will also frequently lead to a more parsimonious model than selection based on p-values since it chooses variables by relevance to the exposure-outcome association, rather than the association with the outcome alone. The approach also extends background-knowledge methods by checking starting assumptions against the data and requiring researchers to justify mismatches or adapt assumptions appropriately. The approach complements the recently proposed method of adjusting on all assumed parents of exposure and outcome [21] as it can incorporate adjustment decisions when parent variables are measured with error and can achieve a more parsimonious model by excluding parent variables which do not lie on biasing pathways. Of course, sensitivity analyses to explore the impact of possible unmeasured confounding [53] remain important.

An important point concerns the possibility of incidental cancellations and small effects. Finding a meaningful difference in the add-one pattern for a variable *when no difference is implied by the DAG* indicates the need to review the variable’s relationships. However, finding no meaningful difference in the add-one or minus-one patterns *when a difference is implied* is not, strictly speaking, inconsistent with the DAG. This is because of the possibilities of incidental cancellations across pathways and of changes which simply do not exceed the pre-defined meaningful threshold. For this reason, we suggest that the researcher maintain such arrows (thereby assuming “weak faithfulness” rather than faithfulness (see [32] p.190), but label these arrows for other research teams to examine with different datasets.

A potential criticism of the approach is that it does not eliminate background knowledge from adjustment-variable selection. Indeed, the examples include instances of needing background knowledge to distinguish between DAGs giving the same add-one and minus-one patterns (e.g. confounding- vs. mediating-pathway examples, measurement-error vs. bias-amplification examples). It is well known that different DAGs can imply the same statistical relationships [3,7,54], making an appeal to background knowledge unavoidable when using DAGs in applied work. We do not consider this a limitation, however, seeing background knowledge as valid information which should rarely be over-ruled by any single dataset but, rather, reviewed in light of the patterns in the data. This is particularly appropriate in clinical epidemiology, where we frequently know quite a lot about likely relationships between variables. In contrast, the approach is unlikely to be well adapted to datasets for which researchers have very little background knowledge, when alternative approaches such as DAG-discovery algorithms (below) may be used.

Another potential criticism is that the approach only addresses variable relationships relevant to the effect of interest, remaining agnostic about other regions of the DAG. This aims to focus on the research question at hand and to minimize the risk of “getting lost” in trying to explore all possible associations in the DAG, many of which do not directly impact on the selected exposure-outcome estimate. A researcher wishing to explore the full DAG could apply a DAG-discovery algorithm (e.g. the PC, GES, or FCI algorithms; see the TETRAD project’s website and [7]). Such algorithmic approaches use statistical tests or scoring rules to identify edges between variables and can incorporate background knowledge such as the temporal ordering of variables or the forced inclusion or exclusion of arrows. However, they have proven controversial [8] and have not yet crossed over into applied epidemiologic research. Nonetheless, recent applications of these algorithms in the biomedical literature for data with many variables and little background knowledge have been interesting [55]. In the approach proposed in this article, a researcher could use these algorithms to explore additional prior starting DAGs. In our experience, however, there are challenges to using these algorithms currently, including handling datasets with mixed continuous and categorical variables and dealing with issues such as measurement error and bias amplification.

We wish to highlight several additional limitations of the proposed approach. Like the change-in-estimate procedure, the approach is *ad hoc* and informal as it depends on arbitrary thresholds and is not founded on well-defined statistical tests with appropriate theoretical properties. In addition, as discussed above, different DAG structures can give the same implied add-one and minus-one patterns and so more than one DAG will be consistent with the observed patterns. For this reason, the researcher should present all identified DAGs with implied patterns consistent with those observed; further, researchers should always remember that other DAGs (not identified) will also be consistent with the patterns.

Several extensions to the approach are possible, should it appeal to epidemiologists working on applied questions. These include how best to address sampling variability in the patterns, comparing the performance of different rules based on the proportion of bootstrap samples which fall outside the meaningful threshold. Another potential extension concerns precision in choosing the adjustment set. We note that a researcher may wish to adjust on additional variables to improve precision [56] and may wish to delete variables from the final adjustment set based on precision of estimates, as concluded in [6]. Researchers should of course bear in mind that, as with any *a posteriori* variable selection, estimates from a revised DAG will tend to be over-precise. Finally, it may be possible to extend the approach to include recent advances in DAG theory, including selection variables to encode differences between populations (and so uncertainty about arrows) [57], signed DAGs which specify assumptions about the positive or negative direction of paths [58], and interactions using sufficient causation DAGs [59].

## Conclusions

In summary, we have proposed a novel approach to adjustment-variable selection in epidemiology which combines existing knowledge-based and statistics-based methods. It requires a researcher to present background-knowledge assumptions in a DAG, to compare these against patterns in the data, and to review assumptions accordingly. It also ensures clear communication of assumptions and uncertainties to other researchers and readers in a standardized graphical format. As the approach requires background knowledge, it is probably best suited to areas such as clinical epidemiology where researchers know quite a lot about *a priori* plausible variable relationships. Researchers can use this approach as an additional tool for selecting adjustment variables when analyzing epidemiological data.

## Competing interests

The authors declare that they have no competing interests.

## Authors’ contributions

DE, BC, and AF conceived the idea through their interests in confounder selection and directed acyclic graphs. CV and TL were responsible for the peritoneal dialysis data and contributed to the development and interpretation of the empirical example. DE did the analyses and drafted the manuscript. All authors critically reviewed the drafts and approved the final version.

## Pre-publication history

The pre-publication history for this paper can be accessed here:

http://www.biomedcentral.com/1471-2288/12/156/prepub

## Supplementary Material

Additional file 1 (Reviewing a DAG when implied and observed patterns are incompatible; Additional information on the empirical example; Sample R code for the add-one and minus-one graphs). Click here for file

Additional file 2 (Figures containing DAGs as Powerpoint slides). Click here for file
