# Supplementary material for: Combining directed acyclic graphs and the change-in-estimate procedure as a novel approach to adjustment-variable selection in epidemiology
Source: BMC Med Res Methodol. 2012 Oct 11;12:156. doi: 10.1186/1471-2288-12-156 (PMC3570444; doi:10.1186/1471-2288-12-156)
Supplement: Additional file 1 — (Reviewing a DAG when implied and observed patterns are incompatible; Additional information on the empirical example; Sample R code for the add-one and minus-one graphs). [file 1471-2288-12-156-S1.docx]

# WEB APPENDIX

# Reviewing a DAG when implied and observed patterns are incompatible

This section presents an approach to reviewing a DAG because of inconsistent implied and observed patterns. Note that these steps work from the prior DAG, assuming that the DAG is the best possible prior approximation to the unknown best working DAG.

Take a prior DAG with a putative minimal adjustment set S; variables, Z_A_, which do not match the implied add-one pattern; and variables Z_M_, which do not match the implied minus-one pattern. By definition, Z_A_ are not contained in S and Z_M_ are contained in S. Then:

- Write out each path in the DAG from A to Y, connected to A but not to Y, and connected to Y but not to A. Include the path A🡪Y. Show measurement error in the DAG as descending variables on paths;
- Draw a square around each variable in S to show conditioning on these variables;
- For each variable in S, reconsider if it could plausibly have been measured with error or if it is not in fact better represented as a proxy variable for an unmeasured variable. If so, revise the involved variables in S to show this relationship on each path;
- Label each path as open or closed unconditionally and conditional on S, considering variables measured with error as unblocked (as partially conditioned on);
- Remove the arrows entering and leaving each variable in Z_M_. These should be replaced in the final revised DAG, noting them as empirically unsupported in this particular dataset;
- Considering each path in turn, address the questions outlined in the table below based on the status of the path unconditionally and conditional on S;
- If there are unblocked or partially blocked paths after conditioning on S or if there is plausible unmeasured confounding or other residual biases, consider if any variables in Z_A_ could be instrument-like. Consider if a variable in Z_A_ lies on the same path as a variable in S, when the variable in Z_A_ may have an instrument-type effect.

The researcher needs to assess the plausibility of each possibility when reviewing the DAG and then rerun the steps in the article on the revised DAG. The researcher should also consider misspecification of the model and undertake model checking.

| ***For each path connecting A to Y, record if it open or closed conditional on S and unconditionally. Then match this pattern to the X’s in the left-most columns and consider the questions on the matching line.***  ***E.g. if the path connects A to Y, is closed conditional on S and open unconditionally, consider the 3^rd^ line in the top half of the table below.***  ***E.g. If the path does not connect A to Y, when connecting the end variables on the path to A or Y, the path would be open conditional on S and open unconditionally, consider the questions in the 1^st^ line in the bottom half of the table below.*** | | | | | |
| --- | --- | --- | --- | --- | --- |
| ***IF THE PATH CONNECTS A to Y:***  ***Is the path*** | | | | **Questions to consider** | **What for change-in-estimate** |
| **Open** | **Closed** | **Open** | **Closed** |  |  |
| **Conditional on S** | | **Unconditionally** | |  |  |
|  |  |  |  |  |  |
| X |  | X | X | Could any variable in Z_A_ have an ascending or descending relationship with any of the variables on the path which are not in S?  Could any variable in Z_A_ have a confounding, mediating, or collision relationship between any of the variables on the path which are not in S? | Adding the variable in Z_A_ with this relationship would partially block the path, causing a change in estimate.  There is an additional path joining A to Y not in the DAG. Adding the variable in Z_A_ would block this path, causing a change in estimate. |
|  | X |  | X | Could any single variable in Z_A_ have a descending relationship with all of the colliders on the path which are not in S? | Adding the variable in Z_A_ would partially open the path, causing a change in estimate. |
|  | X | X |  | Could any variable in Z_A_ have a confounding, mediating, or collision relationship with any of the variables on the path not in S and lying between the variable in S closest to Y and Y?  (e.g. in A🡨C_1_🡪C_2_🡪C_3_🡪C_4_🡪Y, could the variable in Z_A_ have an association with C_3_ or C_4_?) | There is an additional path joining A to Y not in the DAG. Adding the variable in Z_A_ would block this path, causing a change in estimate. |
|  | X |  | X | Could any variable in Z_A_ have a confounding, mediating, or collision relationship with any of the variables on the path not in S and lying between the variable which closes the path closest to Y and Y?  (e.g. in A🡨C_1_🡪C_2_🡨C_3_🡪C_4_🡪Y, could the variable in Z_A_ have an association with C_3_ or C_4_?) | There is an additional path joining A to Y not in the DAG. Adding the variable in Z_A_ would block this path, causing a change in estimate. |
| ***IF THE PATH DOES NOT CONNECT A to Y:*** | | | | **Questions to consider** | **What for change-in-estimate** |
| **If the path were connected to A or Y*,**  **path would be** | | | |  |  |
| **Open** | **Closed** | **Open** | **Closed** |  |  |
| **Conditional on S** | | **Unconditionally** | |  |  |
|  |  |  |  |  |  |
| X |  | X | X | Could any of the variables in Z_A_ have a confounding, mediating, or collision relationship with any of the variables on the path which are not in S? | There is an additional path joining A to Y not in the DAG. Adding the variable in Z_A_ would block this path, causing a change in estimate. |
|  | X | X |  | Could any of the variables in Z_A_ have a confounding, mediating, or collision relationship with any of the variables on the path not in S and lying between the variable in S closest to Y and Y? | There is an additional path joining A to Y not in the DAG. Adding the variable in Z_A_ would block this path, causing a change in estimate. |
|  | X |  | X | Could any of the variables in Z_A_ have a confounding, mediating, or collision relationship with any of the variables on the path not in S and lying between the variable which closes the path closest to Y and Y? | There is an additional path joining A to Y not in the DAG. Adding the variable in Z_A_ would block this path, causing a change in estimate. |

* consider connecting the path’s end variable to A or Y with an undirected arc, e.g. a path of C_1_🡪C_2_🡪Y would be imagined as A—C_1_🡪C_2_🡪Y.

# Additional information on the empirical example

The empirical example uses data from the French Language Peritoneal Dialysis Registry (Registre de Dialyse Péritonéale de Langue Française (RDPLF)). A time-to-event analysis of these data on a larger patient sample can be found in [1]. The example here is purely illustrative, aiming to demonstrate the proposed approach to covariable selection using real data.

We analyzed 1,153 non-diabetic patients who started peritoneal dialysis (PD) in metropolitan France and Belgium between January 1, 2002 and December 31, 2003. Exposure was defined as an underlying nephropathy of polycystic kidney disease (PKD) (n=95 (6.9% of all patients)). The outcome was defined as either *dead* or *not dead* 5 years after the start of PD (n=393 deaths (34.1%); n=17 (17.9%) in the PKD group; n=376 (35.5%) in the other nephropathy group). Other variables extracted from the RDPLF database were sex, age and Charlson Comorbidity Index at PD initiation, and type of PD and type of assistance at day 90 after PD initiation (the standard measure in the dialysis literature). We estimated the RD and 95% confidence intervals (95%CI) by linear models with robust variances from generalized estimating equations [2].

We would like to mention two features of the analysis. Firstly, in comparing patients with PKD with patients with other nephropathies, we note that the comparison group includes a range of non-PKD nephropathies. It is therefore similar to a situation of compound treatments [3], the net association depending on the proportions of the other nephropathies in the comparison group. Secondly, we note that we did not separate out the competing risks in the analysis (PD patients can experience death, transfer to haemodialysis, and kidney transplantation). Rather, we included in the *not dead* group those patients experiencing any outcomes other than death, so as to provide a binary outcome for illustrative purposes.

1. Lobbedez T, Touam M, Evans D, Ryckelynck J-P, Knebelman B, Verger C. Peritoneal dialysis in polycystic kidney disease patients. Report from the French peritoneal dialysis registry (RDPLF). Nephrology Dialysis Transplantation. 2011;26:2332–2339.

2. Cheung YB. A modified least-squares regression approach to the estimation of risk difference. American Journal of Epidemiology. 2007;166:1337–1344.

3. Hernán MA, VanderWeele TJ. Compound treatments and transportability of causal inference. Epidemiology. 2011;22:368–377.

# Sample R code for the add-one and minus-one graphs

*Code for the non-parametric bootstrap of the add-one and minus-one changes is available from the authors on demand.*

# SAMPLE R-CODE FOR THE ADD-ONE AND MINUS-ONE PLOTS

# Note: The dataframe must include only variables shown on the DAG.

# Code is for continuous and binary exposures but can be readily adapted to

# categorical exposures with >1 grouping.

# Define exposure & outcome

exposureVariable <- "..."

outcomeVariable <- "..."

# Define putative minimally sufficient adjustment set

variablesMAS <- c(

"...",

"...",

"..."

)

# Define meaningful change threshold

threshold <- ...

# Run add-one and minus-one regressions

allVariables <- colnames(…) # include name of dataframe

variablesNotMAS <- allVariables[-which(allVariables %in% c(variablesMAS, outcomeVariable, exposureVariable))]

formulaVariablesMAS <- as.formula(paste(outcomeVariable, " ~ ", paste(c(exposureVariable, variablesMAS), collapse= "+")))

model1 <- glm(formulaVariablesMAS, family='gaussian', data=...) # adapt regression model for chosen association estimate

# Add-one

coefficientsAddOne <- vector(length=(length(variablesNotMAS) + 1))

coefficientsAddOne[1] <- coef(model1)[2]

for (i in seq(along.with=variablesNotMAS)) {

formulaAddOneTemp <- as.formula(paste(c(formulaVariablesMAS, variablesNotMAS[i]), collapse= "+"))

modelAddOneTemp <- glm(formulaAddOneTemp, family='gaussian', data=...) # must match regression in model1

coefficientsAddOne[i+1] <- coef(modelAddOneTemp)[2]

names(coefficientsAddOne) <- c("MAS", variablesNotMAS)

}

# Minus-one

coefficientsMinusOne <- vector(length=(length(variablesMAS) + 1))

coefficientsMinusOne[1] <- coef(model1)[2]

for (i in seq(along.with=variablesMAS)) {

formulaMinusOneTemp <- as.formula(paste(c(formulaVariablesMAS, variablesMAS[i]), collapse= "-"))

modelminusOneTemp <- glm(formulaMinusOneTemp, family='gaussian', data=...) # must match regression in model1

coefficientsMinusOne[i+1] <- coef(modelminusOneTemp)[2]

names(coefficientsMinusOne) <- c("MAS", variablesMAS)

}

# Graph

coefficientsAddOneMinusOne <- c(coefficientsAddOne[-1], coefficientsMinusOne[-1])

par(mfrow=c(4,1), mar=c(0,4,1,2)) # adapt as needed

yLimits <- c(-0.08,0.02) # set manually

plot(coefficientsAddOneMinusOne, pch=16, ylim=yLimits, xaxt="n", las=1, xlab="", ylab="")

abline(h=coef(model1)[2])

abline(h=coef(model1)[2]+c(threshold, -threshold), lty="dotted") # absolute change of threshold

#abline(h=coef(model1)[2]*c(1.1, 0.9)) # plots +/-10% change in estimate if preferred

abline(v=length(coefficientsAddOne) - 0.5)

axis(side=1, labels=names(coefficientsAddOneMinusOne), at=1:length(coefficientsAddOneMinusOne), las=2)

par(font=2)

legend(0.75, yLimits[2]+0.015, legend = "ADD-ONE", bty="n") # adapt placement

legend(length(coefficientsAddOne)-0.58, yLimits[2]+0.015, legend = "MINUS-ONE", bty="n") # adapt placement
