# Supplementary material for: Combining directed acyclic graphs and the change-in-estimate procedure as a novel approach to adjustment-variable selection in epidemiology
Source: BMC Med Res Methodol. 2012 Oct 11;12:156. doi: 10.1186/1471-2288-12-156 (PMC3570444; doi:10.1186/1471-2288-12-156)
Supplement: Additional file 2 — (Figures containing DAGs as Powerpoint slides). [file 1471-2288-12-156-S2.ppt]

## Slide 1
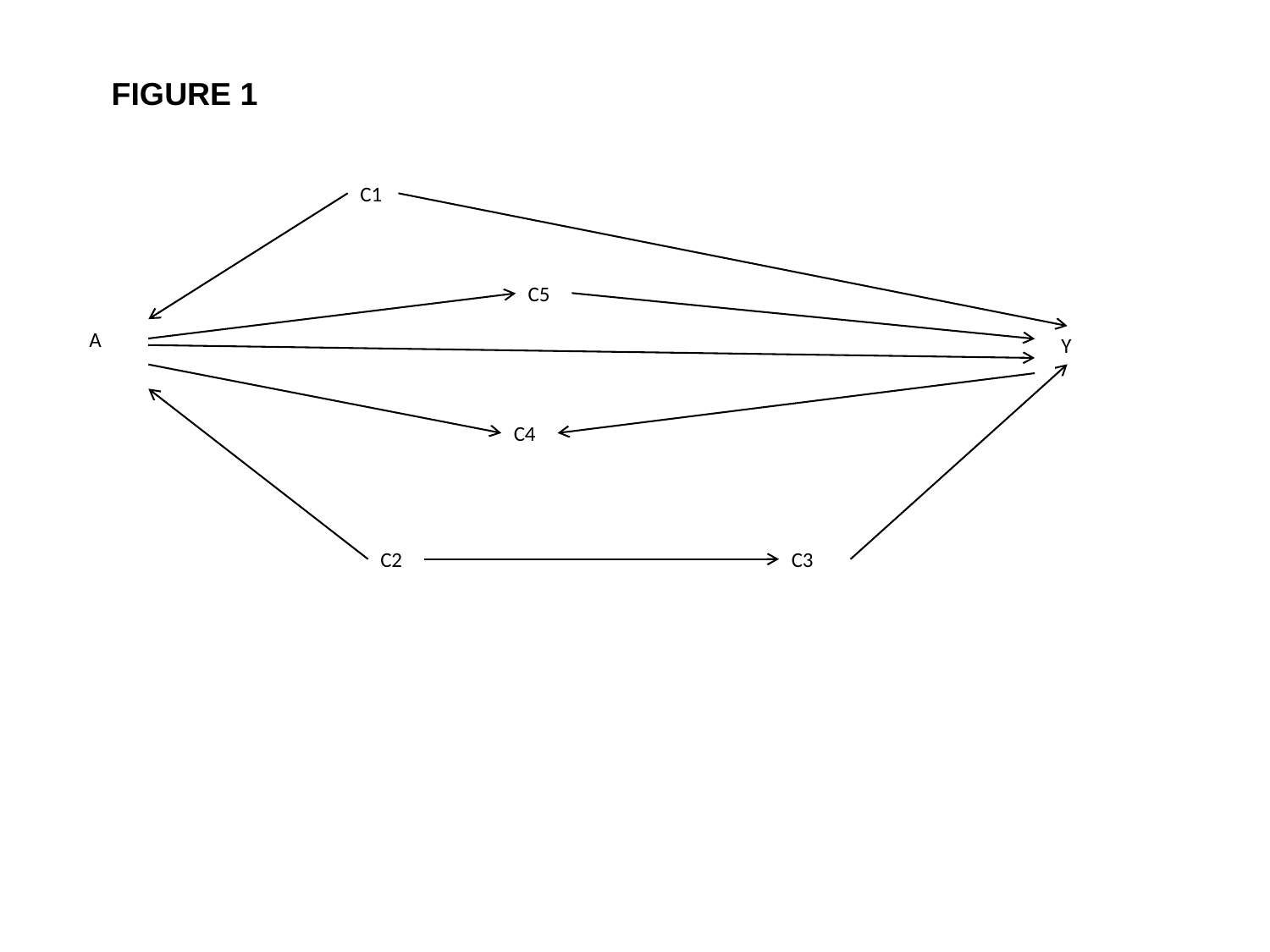

FIGURE 1
C1
C5
A
Y
C4
C2
C3

## Slide 2
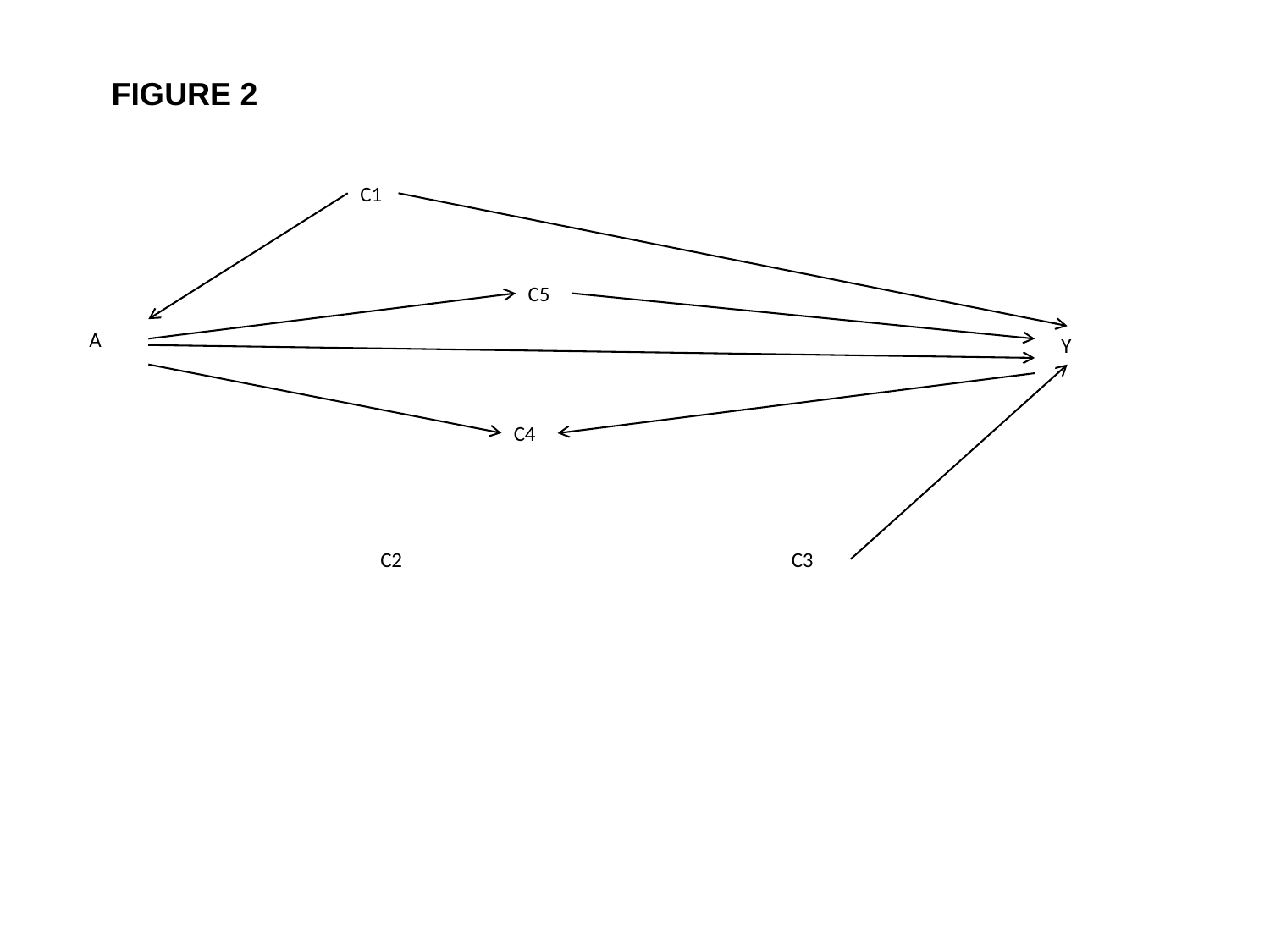

FIGURE 2
C1
C5
A
Y
C4
C2
C3

## Slide 3
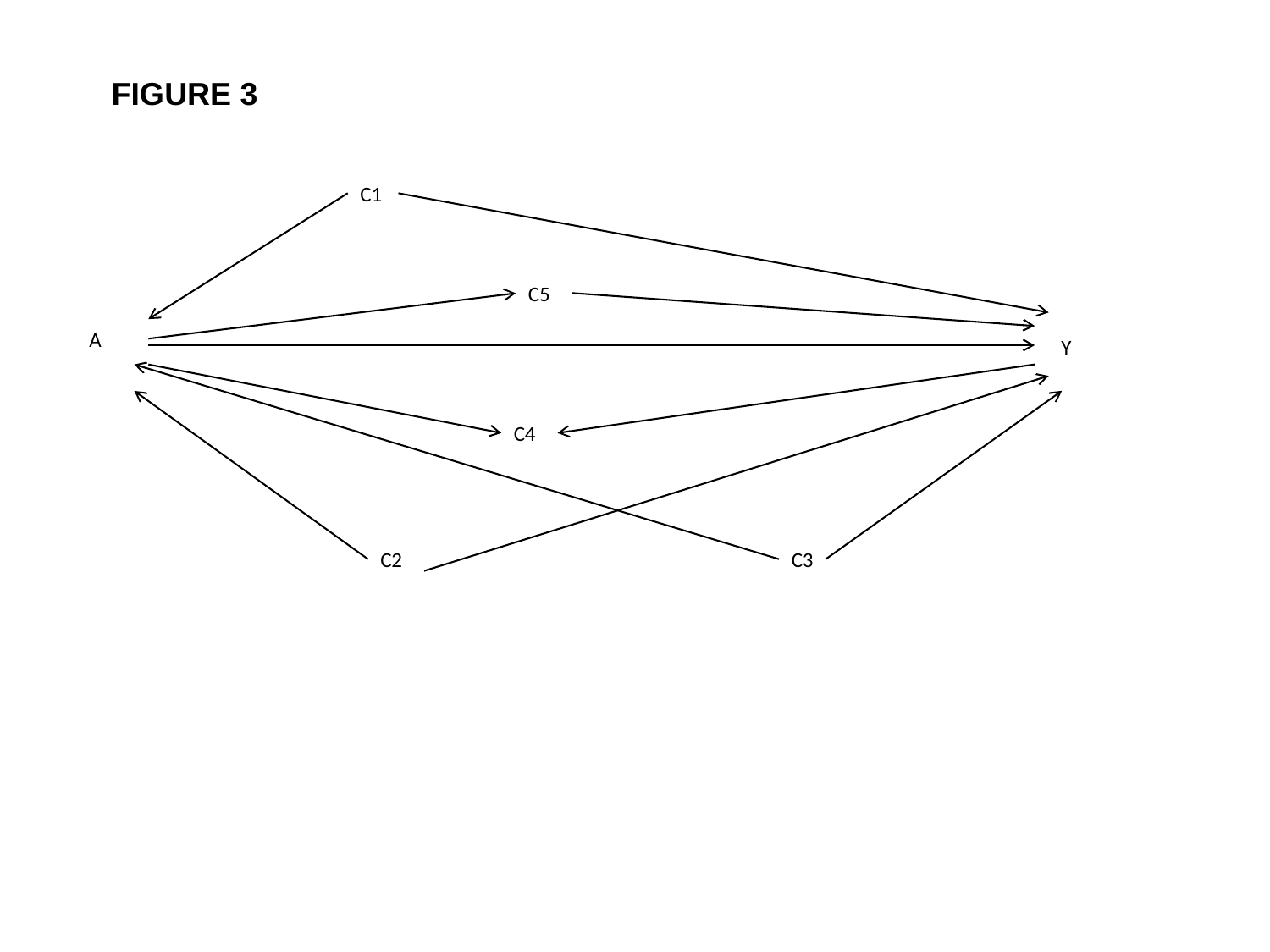

FIGURE 3
C1
C5
A
Y
C4
C2
C3

## Slide 4
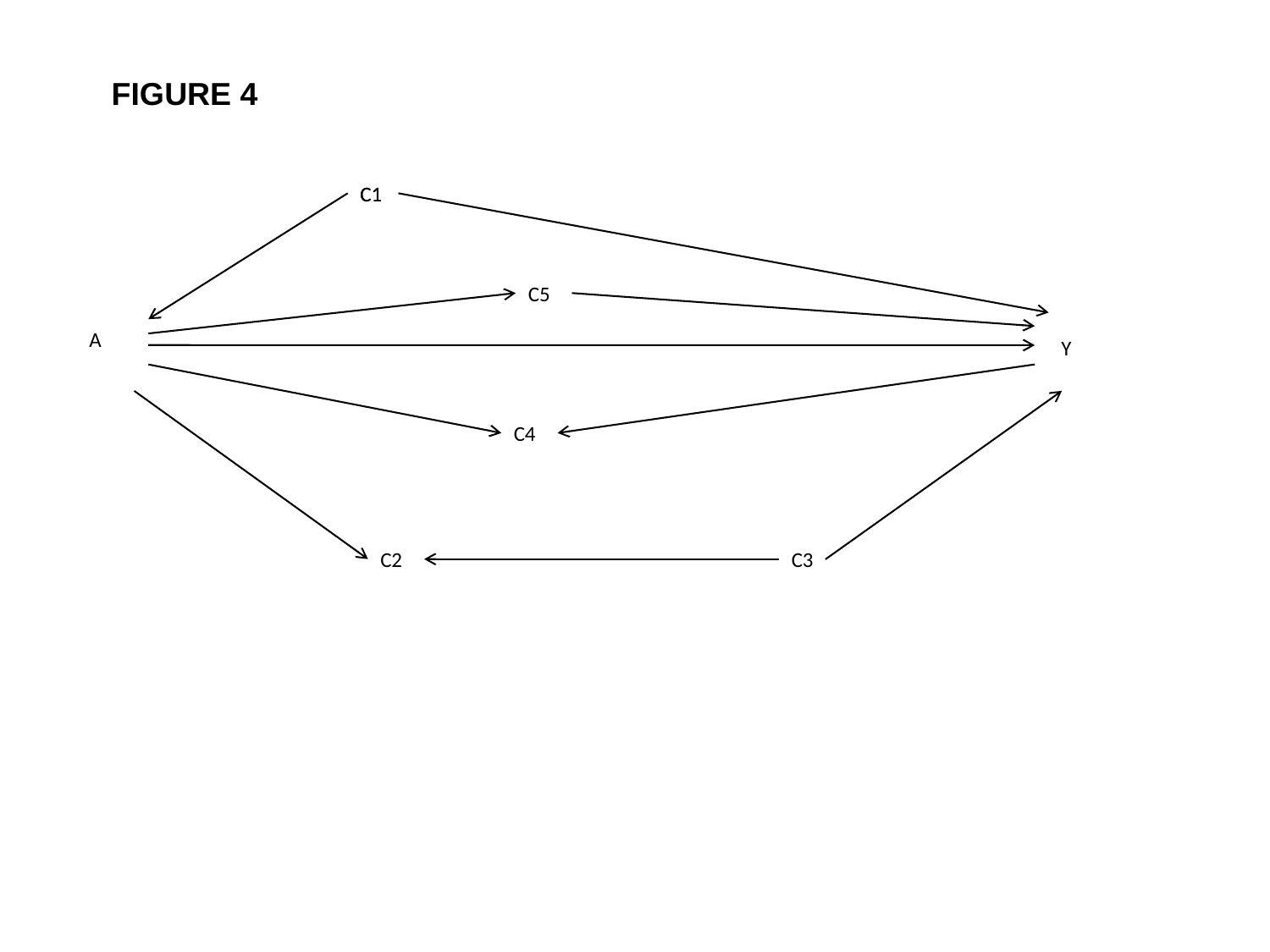

FIGURE 4
C1
C1
C5
A
Y
C4
C2
C3

## Slide 5
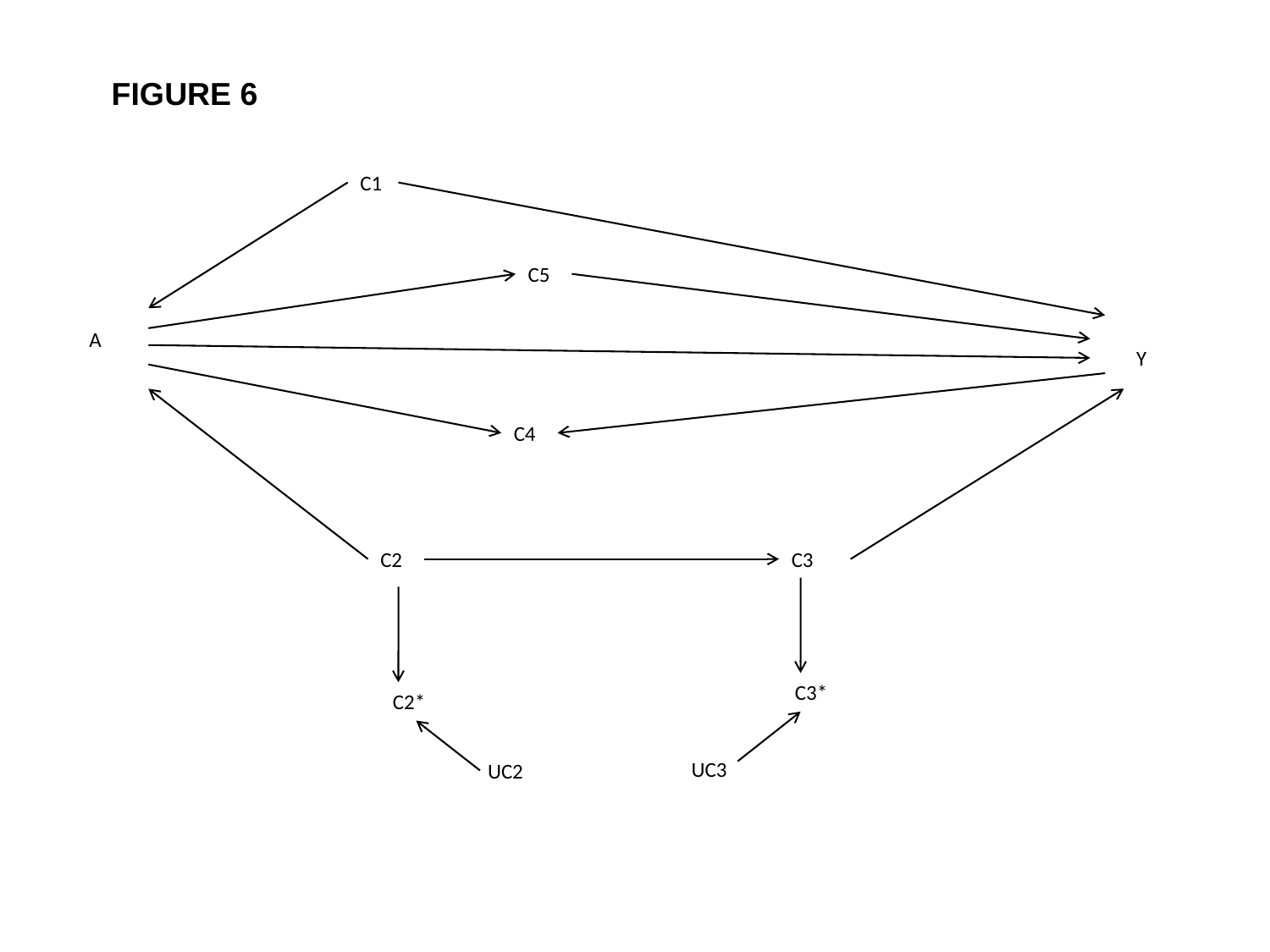

FIGURE 6
C1
C5
A
Y
C4
C2
C3
C3*
C2*
UC3
UC2

## Slide 6
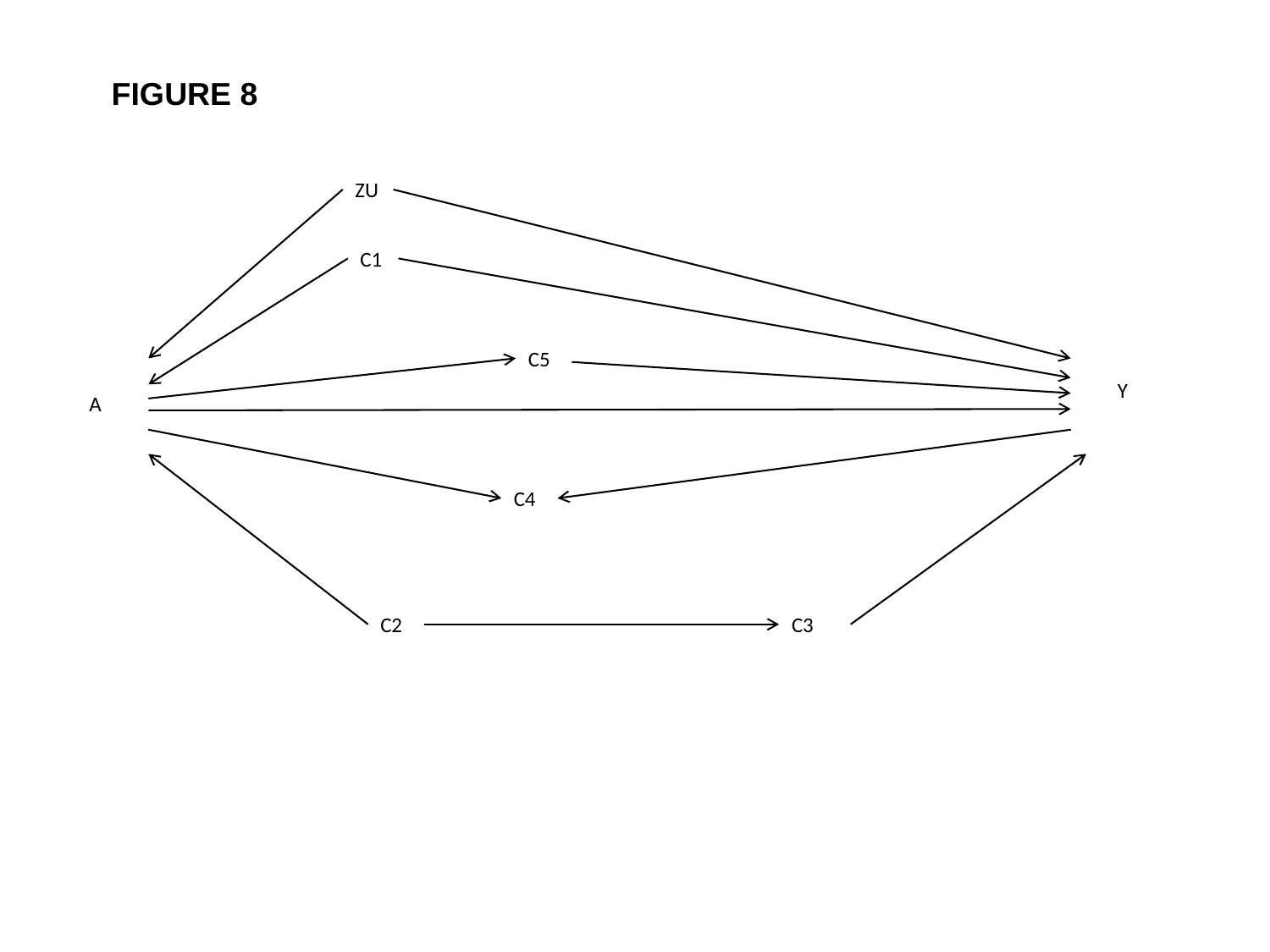

FIGURE 8
ZU
C1
C5
Y
C4
C2
C3
A

## Slide 7
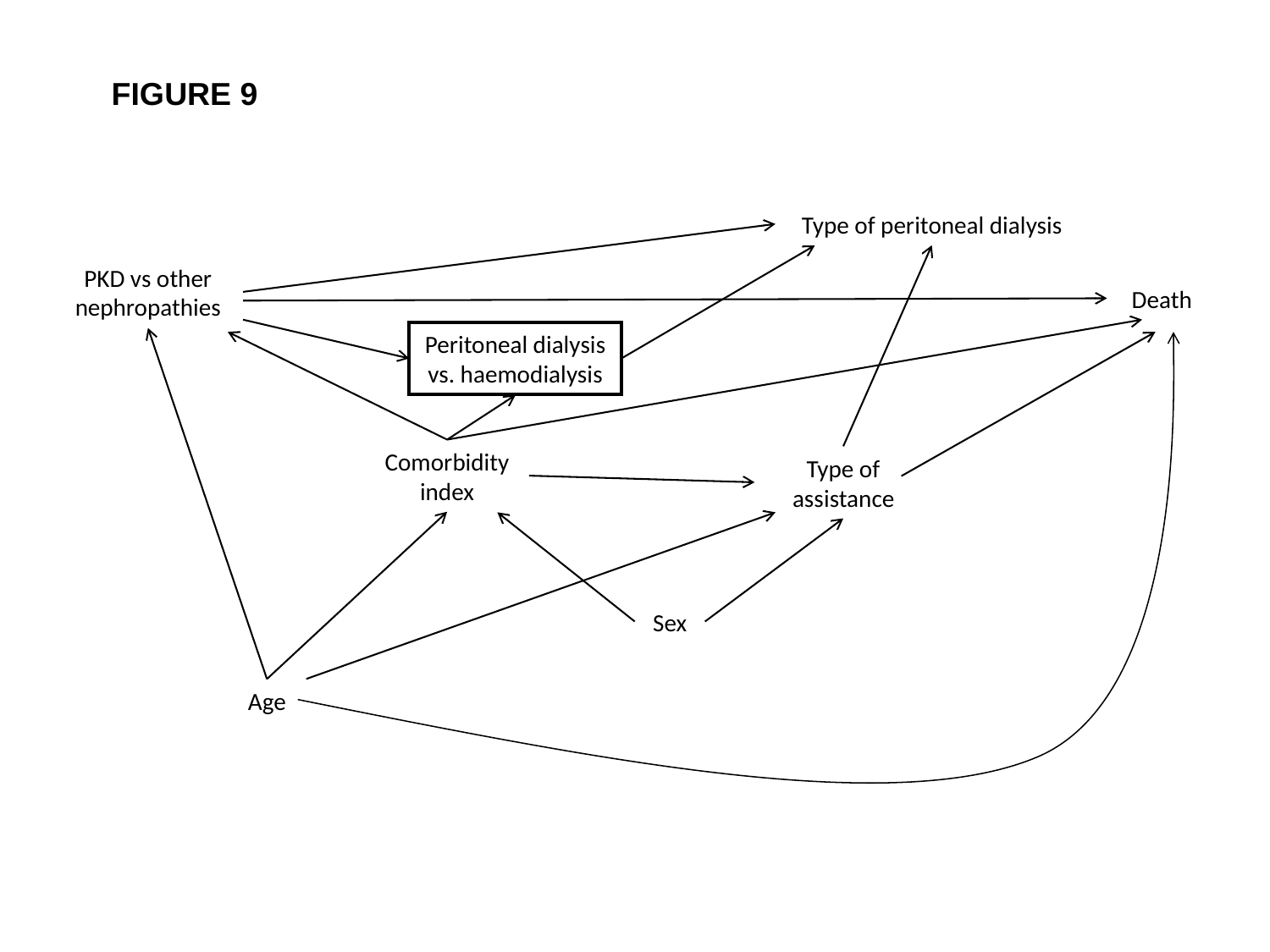

FIGURE 9
Type of peritoneal dialysis
PKD vs other nephropathies
Death
Peritoneal dialysis vs. haemodialysis
Comorbidity index
Type of assistance
Sex
Age

## Slide 8
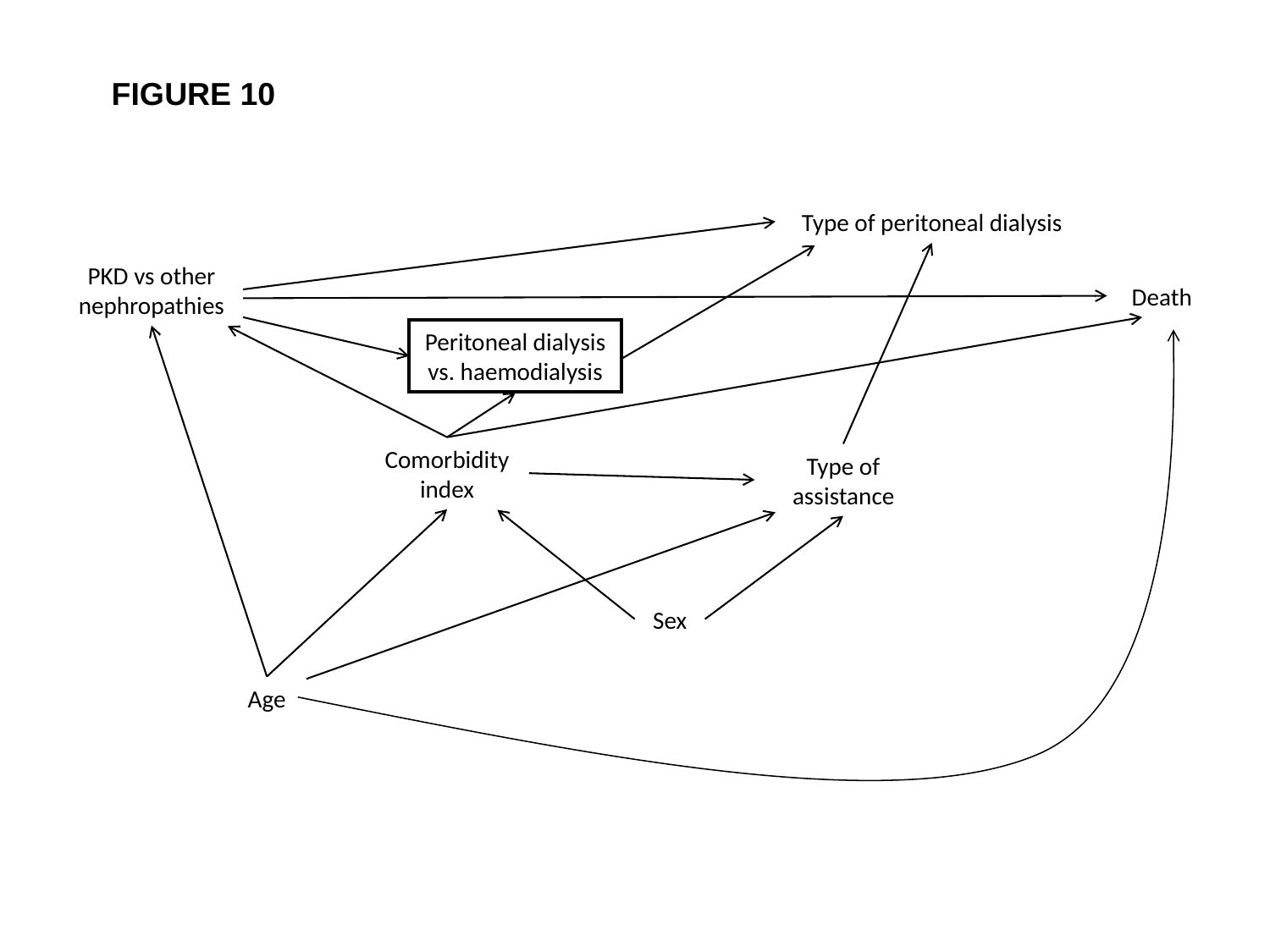

FIGURE 10
Type of peritoneal dialysis
PKD vs other nephropathies
Death
Peritoneal dialysis vs. haemodialysis
Comorbidity index
Type of assistance
Sex
Age

## Slide 9
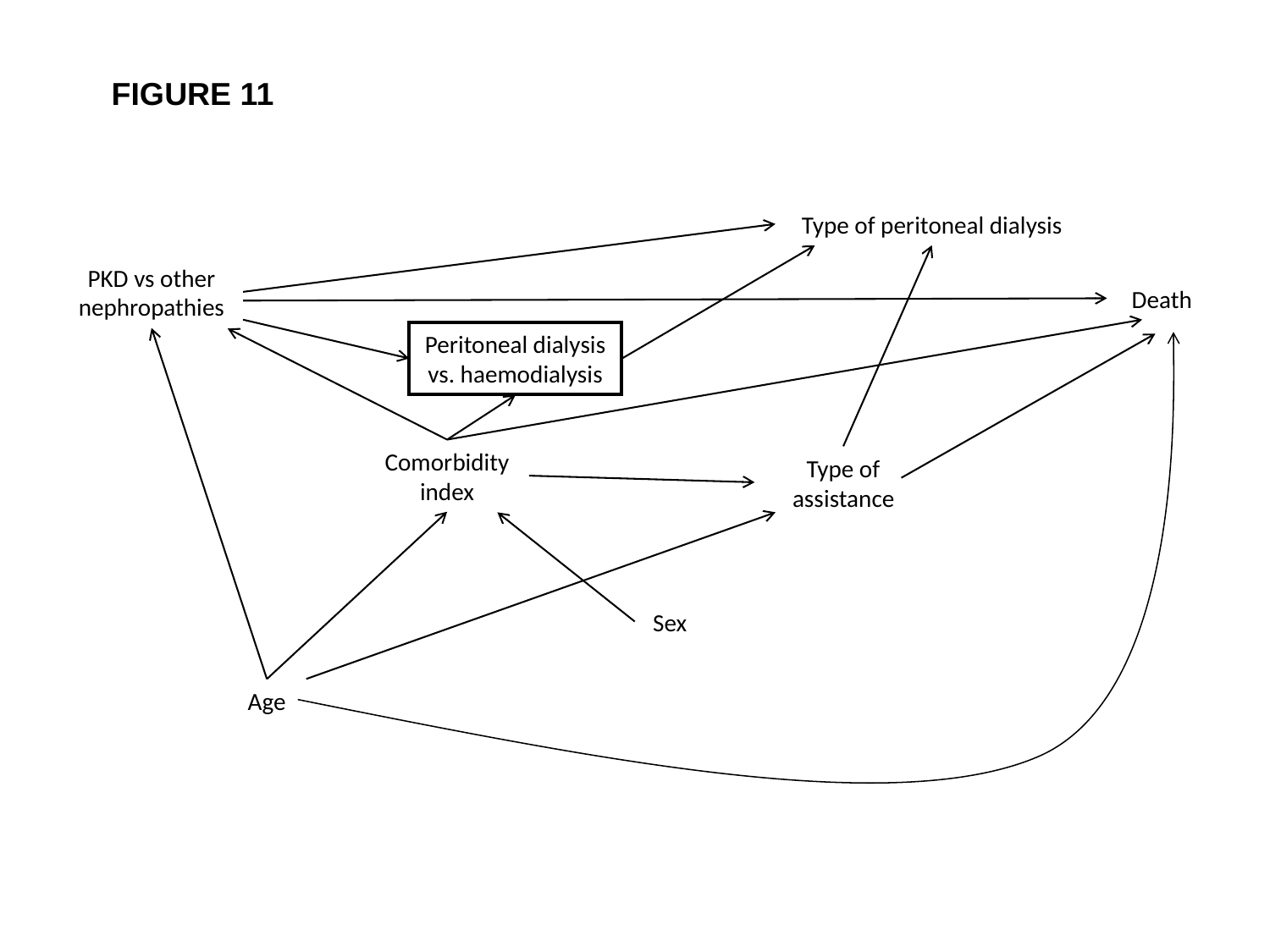

FIGURE 11
Type of peritoneal dialysis
PKD vs other nephropathies
Death
Peritoneal dialysis vs. haemodialysis
Comorbidity index
Type of assistance
Sex
Age

## Slide 10
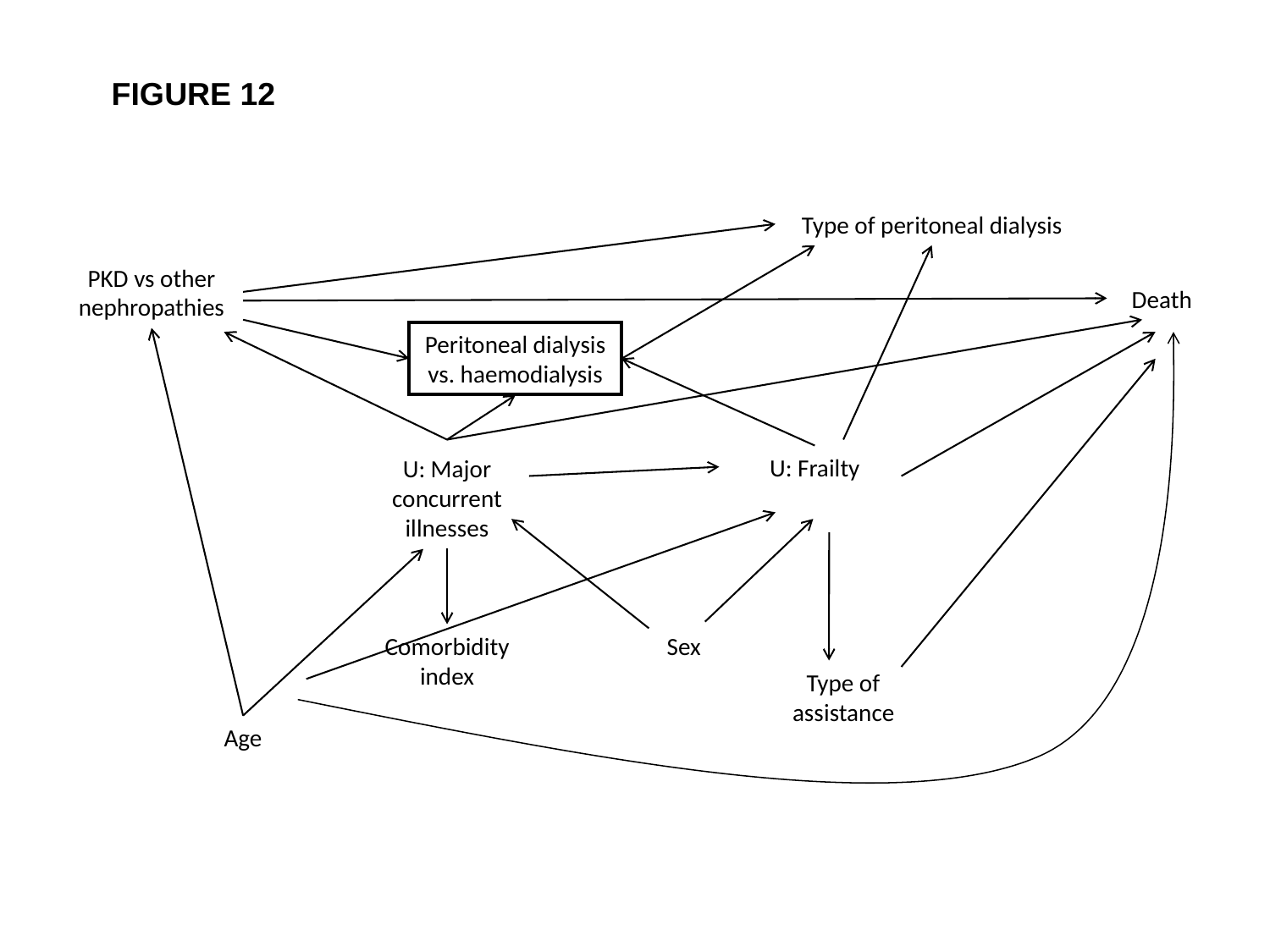

FIGURE 12
Type of peritoneal dialysis
PKD vs other nephropathies
Death
Peritoneal dialysis vs. haemodialysis
U: Major concurrent illnesses
Comorbidity index
Sex
Type of assistance
Age
U: Frailty
